# Adaptable surfactant-mediated method for the preparation of anisotropic metal chalcogenide nanomaterials

**DOI:** 10.1038/s41598-018-21328-7

**Published:** 2018-02-12

**Authors:** S. A. McCarthy, R. Ratkic, F. Purcell-Milton, T. S. Perova, Y. K. Gun’ko

**Affiliations:** 1 School of Chemistry, Trinity College Dublin, Dublin 2, Ireland; 2 CRANN, Trinity College Dublin, Dublin 2, Ireland; 3 BEACON, Bioeconomy Research Centre, University College Dublin, Dublin 4, Ireland; 4 Department of Electronic and Electrical Engineering, Trinity College Dublin, Dublin 2, Ireland

**Keywords:** Materials chemistry, Synthesis and processing

## Abstract

The hot injection synthesis of nanomaterials is a highly diverse and fundamental field of chemical research, which has shown much success in the bottom up approach to nanomaterial design. Here we report a synthetic strategy for the production of anisotropic metal chalcogenide nanomaterials of different compositions and shapes, using an optimised hot injection approach. Its unique advantage compared to other hot injection routes is that it employs one chemical to act as many agents: high boiling point, viscous solvent, reducing agent, and surface coordinating ligand. It has been employed to produce a range of nanomaterials, such as CuS, Bi_2_S_3_, Cu_2-x_Se, FeSe_2_, and Bi_4_Se_3_, among others, with various structures including nanoplates and nanosheets. Overall, this article will highlight the excellent versatility of the method, which can be tuned to produce many different materials and shapes. In addition, due to the nature of the synthesis, 2D nanomaterial products are produced as monolayers without the need for exfoliation; a significant achievement towards future development of these materials.

## Introduction

Advances in nanotechnology and materials science has enabled the control of the dimensions and properties of materials at the nanoscale. Interesting variations in their properties based on differences in their size and shape is expected. These morphological variations alter how particles interact with interfaces and 3D chemical structures. In addition, structural anisotropy allows nanoparticles of larger dimensions while retaining nano-related properties, due to quantum confinement in one or more directions. Since the degree of confinement in one direction is frequently different from those in the other directions, the energetic properties of anisotropic nanostructures can significantly differ from those of the isotropic systems. These anisotropic nanostructures are of great interest for science and technology as they are expected to demonstrate considerable advantages over their isotropic counterparts in various areas, including enhanced energy harvesting, improved charge transport properties, nonlinear optical responses, enhanced optical gain and polarized light absorption and emission, among others^1,2^.

The crystallographic structure of a solid material, its surface free energy, surfactants, various templates, kinetically controlled by supersaturation and other factors all play crucial roles in the anisotropic growth of nanomaterials^3–5^. Thermal decomposition techniques such as the hot injection method is one of the most widely used synthetic approaches for nanomaterial synthesis, due to its ability to form highly crystalline, monodisperse nanoparticles in reasonable yield^6–10^. However, there is a scarcity of adaptable methods for the shape-selective synthesis of anisotropic particles, because the underlying chemistry of each system is a deciding factor^5^.

The shape of anisotropic particles are determined by competing factors, such as preferential attachment to different crystal facets, surfactant-mediated directional growth, and surface coordinating ligands^11–13^. There have been a number of interesting anisotropic nanostructures prepared using this technique including nanorods^14–16^, wires^17,18^, tetrapods^19–21^, multipods^22^, dumbbells^23^ and platelets^24,25^, among others. However, most of the methods reported to date, describe only the synthesis of one material and have specific cocktails of many various reagents.

Surfactant-assisted growth of nanoparticles, using the hot injection approach is a fundamental chemical strategy in wet chemical techniques. It is a large and diverse field of chemistry. Variation comes from the selection of solvents, surfactants, ligands, precursors, combinations of these, and also distinctions in the many factors comprising the reaction conditions. The possibilities are virtually endless. In most cases, the strategy involves one or more high boiling point organic solvents, one or more reducing agents, one or more coordinating ligands, and a diverse range of chemical precursors. It is also a complex, multifaceted field requiring understanding and collaboration across a number of disciplines.

In this manuscript we present an adaptable hot injection approach. The key advantages of oleylamine (OAm) over other solvents are its ability to act as many different agents, including high boiling point, viscous solvent, reducing agent and coordinating ligand *via* terminal amine. This approach employs finely tuned concentration, temperature and precursors, and an optimised purification strategy, for the synthesis of various anisotropic chalcogenide nanomaterials. In addition NMP is used to facilitate hot injection of the metal precursors. NMP also shows great versatility. It has a high boiling point, amenable to the reaction conditions, it can effectively dissolve or disperse a wide range of metallo-organic compounds and metal salts, including water soluble salts, transferring them to the organic OAm phase, and its chemical inertness prevents side reactions occurring during the reaction. The key advantages of this method are its economy of reagents, elimination of unnecessary reagents, reproducibility, versatility and scalability.

The materials explored in this article have many diverse and useful applications^26–28^. Material specific examples of these properties and applications are shown in Table 1. However, the list is not exhaustive and is intended merely as a guide to readers towards literature specific to the materials. In addition, OAm has been used as a reagent in a wide variety of reactions in combination with other reagents, for the production of both isotropic and anisotropic materials^11^. Overall, the simple strategy presented here, involving an economy of reagents and similar conditions, formed the basis for the production of anisotropic materials for a variety of semiconductors. There is also scope to apply these methods to other materials, including semiconductors, metals, and metal oxides.

**Table 1:** Key applications of selected metal chalcogenide nanomaterials.

NANOMATERIAL	APPLICATION/PROPERTIES
Copper Sulfide	Plasmonics/Fluorescence^38,46,47^ Photothermal therapy^48^
Bismuth Sulfide	Thermoelectrics^49,50^, Photovoltaics^51^, Photocatalysis^52^, X-ray computed tomography imaging^53,54^
Copper Selenide	Plasmonics^38,55^, Photothermal therapy^56^, Optoelectronics^57,58^, Energy^59^
Iron Selenide	Superconductivity^60–62^, Photoconductivity^42^,
Bismuth Selenide	Thermoelectrics^63,64^, Topological Insulator^65^, Cancer Therapy^65^, X-ray computed tomography imaging^65^, Photocatalysis^66^

## Results and Discussion

**Figure 1:**
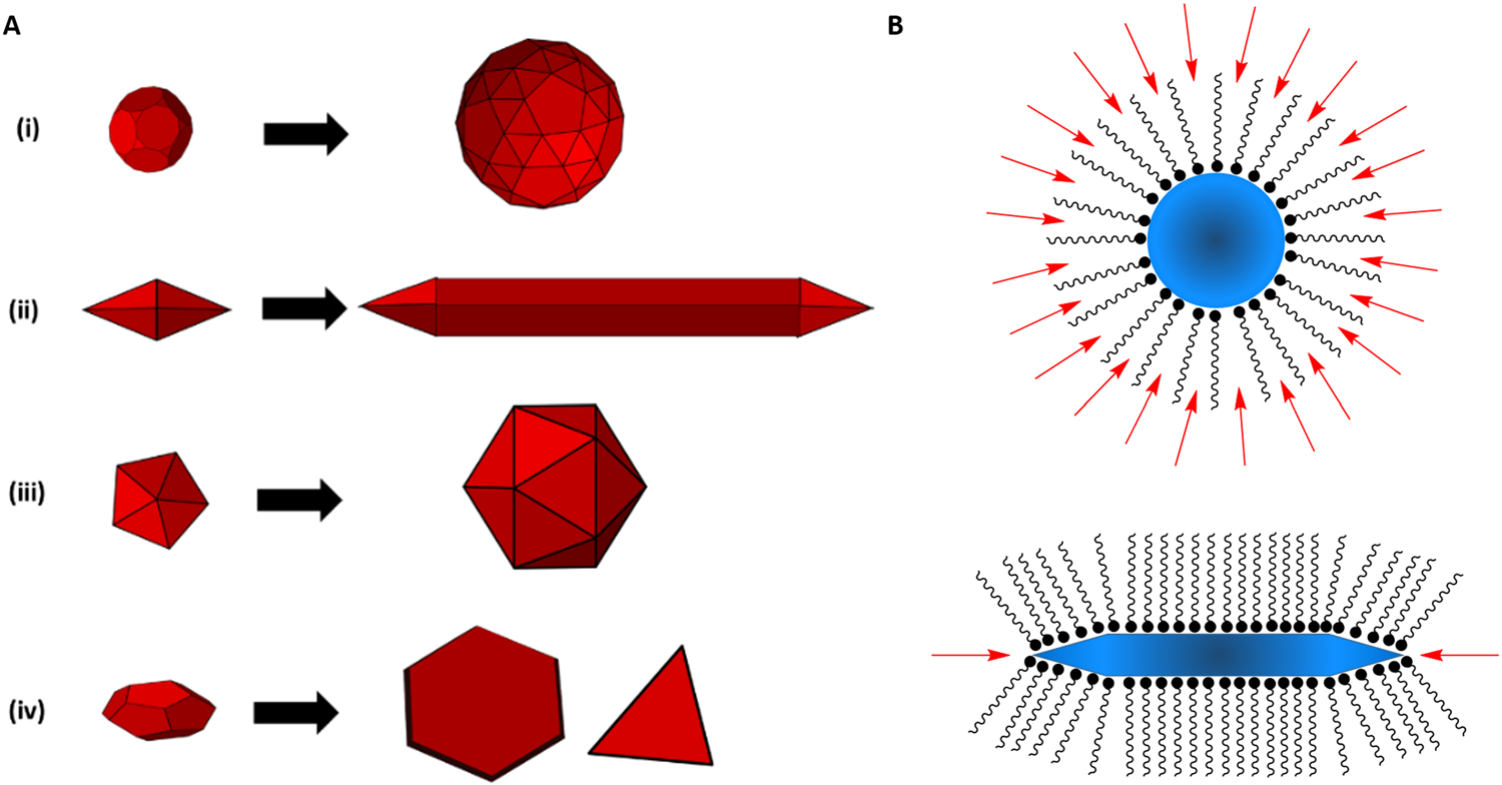
(**A**) Schematic presentation of nanocrystal growth from seeds, with (i) isotropic growth producing spherical particles under thermodynamically favourable conditions, (ii) anisotropic growth from singly twinned seed to produce nanowires or nanosheets, (iii) anisotropic growth from multiply twinned seed to produce icosahedrons, and (iv) anisotropic growth from seeds with stacking faults to produce nanoplates. (**B**) Surfactant-mediated anisotropic growth due to differential stacking along the crystal surface, with high-energy flat facets stabilised by closely stacked surfactant molecules. Crystals are shown in blue, surfactant molecules are shown in black, and preferential attachment points are shown in red.

The rate of crystal formation, depending on the individual crystal dynamics, contributes to anisotropy. In a reaction under thermodynamic control, crystal growth rate is slower and there is unidirectional growth of the different low-energy facets, resulting in an overall rounded shape. This shape has the lowest overall surface energy. However, under kinetic control, crystal growth occurs much faster, and this enhances the difference in surface energy between facets. Thus certain facets grow more quickly than others, resulting in anisotropic shapes^8^. Stacking faults and twinning in the initial crystal seed also play a role in shape. Twinning occurs when crystals grow in opposite directions originating from the same crystal plane. The extent of twinning determines in how many directions crystal growth will occur, and subsequently the shape of the nanoparticle^29,30^. (Fig. 1A) Therefore, temperature is a key consideration for these reactions and small differences in temperature can greatly change the resulting products. Generally reactions were performed over a range of 100–250 °C.

Binding strength of the surfactant coordinating group to the surface of the growing crystal is also important for anisotropic growth and amino groups, such as those in oleylamine, have been used in a number of different methods to promote this type of growth^31,32^. Thus temperature also plays a role in surface stabilisation and accessibility, as it affects the binding strength of the coordinating ligand and therefore reaction kinetics. Amino groups have reasonable binding strength to various metals, and other elements, thus making this synthetic route and methodology more amenable to adaptation for different nanomaterials. Surfactant stacking can also play a role in directed growth of the nanocrystal. (Fig. 1B).

Metal sulfide and selenide reactions reported here proceed firstly by oleylamine reduction of the chalcogen. Anionic sulfur and selenium later react with the cationic metal to form the semiconductor crystals. In the reduction reactions the elemental chalcogen is added to degassed OAm, under inert atmosphere prior to addition of the metal salt. The chalcogen is also added in excess relative to the metal salt, to help drive the reaction and reduce side products, such as metal oxides.

Oleylamine reduction of the chalcogen follows two different mechanisms for sulfur and selenium owing to differences in their redox potential and reactivity, however both involve electron donation from the amine. FTIR and Raman spectra for high concentration solutions of the chalcogen in OAm were measured. Proposed mechanisms for these reactions and spectroscopic data are contained within the ESI. Sulfur reduction results in a dimerised, branched chain surfactant with condensed amidine groups^33–35^. From the FTIR spectra we can see a reduction in amino-related peaks and an increase in amidine and branched chain related peaks. Neither the amine of oleylamine or the amidine product of the reduction reaction featured strongly in Raman measurements and carbon-related peaks predominated. Both FTIR and Raman showed that the olefin is not affected in OAm reduction of sulfur.

For selenium solutions, reduction does not occur at low temperature, and the reaction must be heated to at least 180 °C. In this case, selenium coordinates with the amine, which is then converted to the imine. A similar mechanism has been proposed for Cu reduction by OAm^36^. Chain dimerisation does not occur in this reaction. However, a side reaction is present whereby hydrogen selenide reduces the double bond in the carbon chain, oxidising back to Se^0^. From FTIR we can see a reduction in amino-related peaks and an increase in imine-related peaks, and also a reduction in the olefin-related peaks. From Raman we can see the appearance of the imine and reduction of the olefin also^35^.

The combination of EDX, HRTEM and XRD proved particularly useful in elucidating the composition and crystal structure of the products formed. This information was crucial in designing strategies for the formation of well crystalline structures. The elemental ratios in the products, as determined by EDX acted as a guide in determining the crystal structure, as shown by XRD analysis and/or HRTEM. These techniques also aided in the identification of side products of the reactions. We found that the ratio of metal/chalcogen affected the resulting product. We also found a concentration window of 10–100 mM, a temperature window of 120–220 °C, and an excess of chalcogen relative to the metal salt provide the best results with this method for the materials explored here. In addition, individual reactions can be highly sensitive to small changes in these parameters.

Table S1 (ESI) summarises the reaction conditions and results for selected examples of materials which can be produced using this methodology. Next, more detailed descriptions of their preparation, characterization and properties are discussed. In all cases, reactions were performed over a range of times, temperatures and concentrations; hence only selected experimental conditions and results are presented. Additional materials which are currently being developed are included in the ESI.

### Copper sulfide nanoplates

**Figure 2:**
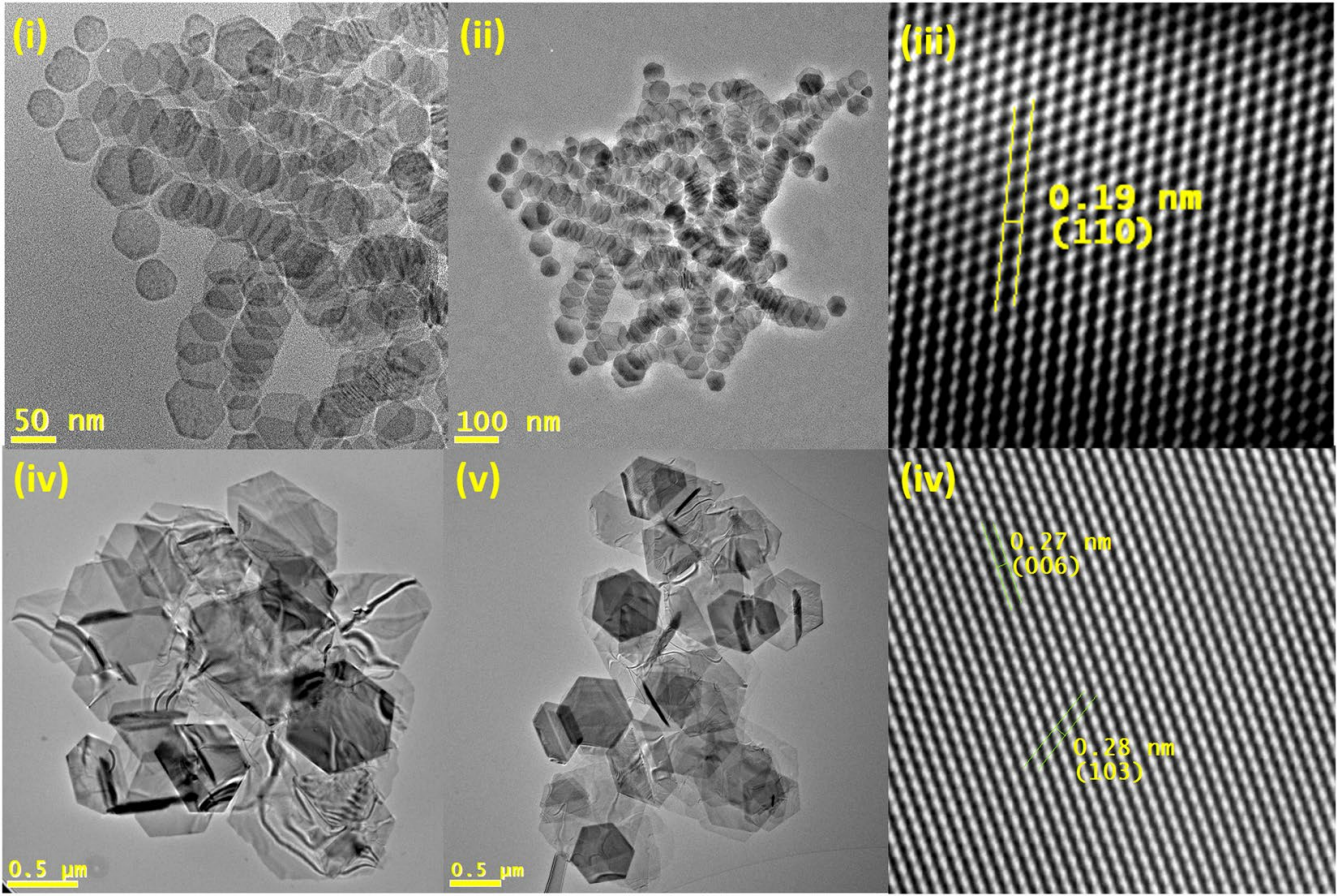
(**i**)–(**ii**) TEM images of small CuS nanoplates produced at 120 °C, (**iii**) masked HRTEM of the small CuS nanoplates from the lateral profile, (**iv**)–(**v**) TEM images of large CuS nanoplates produced at 180 °C and (**vi**) masked HRTEM images of large CuS nanoplates from lateral profile.

In this synthesis, a solution of CuCl_2_.2H_2_O in NMP was added by hot-injection to a solution of reduced sulfur in OAm at various temperatures for 30 min. Two distinct products were obtained at 120 °C and 180 °C. Reaction temperatures in the intermediate region between 120 °C (small nanoplates) and 180 °C (large ultrathin nanoplates) yielded a mixture of the two products, indicating competing reactions. Firstly, small, uniform nanoplates were formed at 120 °C. These nanoplates have a lateral length of 55.5 ± 8.5 nm and width of 4.15 ± 0.25 nm. (Fig. 2) As can be seen from TEM imaging, the plates display an interesting stacking arrangement as a result of their highly uniform size and shape. EDX showed a ratio of 52/48 for Cu/S in this materials and XRD showed the pattern for CuS hexagonal (hex), thus indicating a copper rich surface. (ESI) Lattice fringes for these nanoplates were also visible by HRTEM. From the lateral profile (110) is the dominant phase, with a spacing of 0.19 nm **(**Fig. 2**)**.

**Figure 3:**
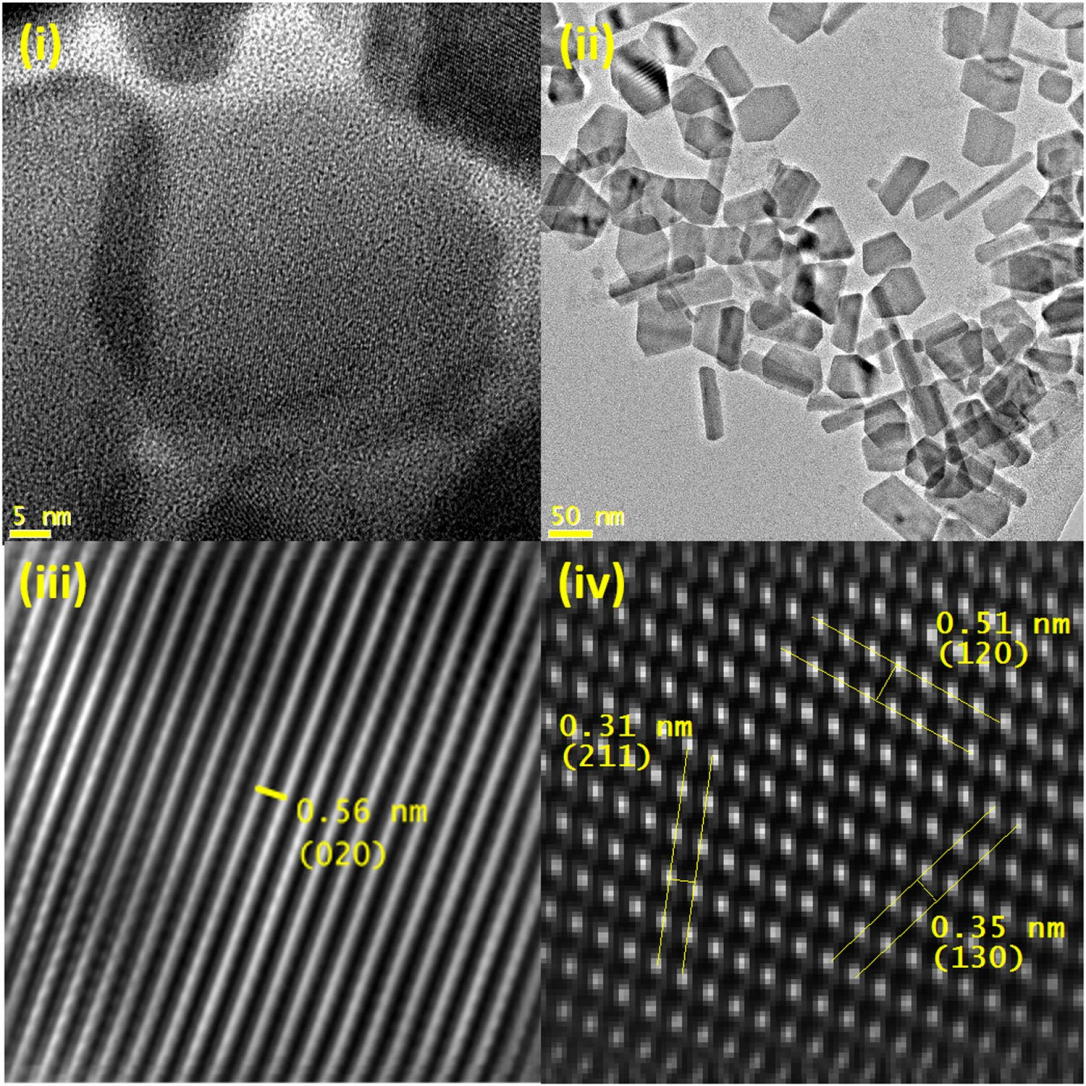
(**i**)–(**ii**) TEM images of Bi_2_S_3_ nanoplates, and masked HRTEM images and measurements for Bi_2_S_3_ nanoplates from (**iii**) axial profile and (**iv**) lateral profile.

At 180 °C, large, ultrathin nanoplates were formed with good uniformity, consisting mainly of hexagons, with a sub-micron lateral length in the region of 755 ± 190 nm and an axial width ≤1 nm, as shown by TEM imaging. **(**Fig. 3**)** XRD analysis showed CuS hexagonal (hex) nanoplates were formed here also. (ESI) Anisotropy is also evident from the high relative intensity of the diffraction point at (006). EDX showed a ratio of 55/45 for Cu/S in this material, indicating a copper-rich surface. (ESI) HRTEM also showed lattice spacings correlating to CuS hexagonal (hex) structure, with fringes at 0.27 nm (006) and 0.28 nm (103) from the lateral profile. **(**Fig. 2**)** We propose that the anisotropic enhancement with increased temperature is due to increased crystal growth kinetics, however this is not a general principal that applies to other materials explored in this work.

UV-IR absorption studies show two peaks for small CuS nanoplates; one of which is associated with band gap absorption taking place below 400 nm, and secondly, a broad peak associated with plasmonic absorption stretching into the infrared which is characteristic of CuS nanostructures, and its peak centred on 1200 nm. (ESI) Large CuS nanoplates showed band gap absorption at 420 nm and also, a large increasing peak stretches into the IR region. (ESI) The copper sulfide plasmon is associated with Cu^2+^ in the lattice, which allow the flow and localization of electrons. Its precise position is a function of Cu^+^ deficiency in Cu_2−x_S crystal and the size/shape of the nanostructure^37^. In addition, low efficiency fluorescence has been reported for these structures particularly for high Cu^+^ content structures^38^, though as expected, none was detected for these nanoplates, as plasmonic interactions dominate.

### Bismuth sulfide nanoplates

In this method, Bi(III)Cl_3_ was used as the hot injection precursor to the solution of reduced sulfur in OAm. Bismuth is highly oxygen sensitive and preparing the hot injection formulation requires more care as a result. In this case the metal salt must be prepared freshly before hot injection. In addition, the precursor does not completely dissolve in NMP and must be added as a fine, well mixed dispersion. If care is taken during degassing, control of inert atmosphere and preparation of the hot injection formulation, the reaction is highly reproducible. After 30 min at 180 °C nanoplates were formed and the product showed excellent uniformity and crystallinity in TEM imaging and analysis (lateral length 67.5 ± 12.8 nm, axial width 10.5 ± 2.8 nm). **(**Fig. 3**)** EDX showed a ratio of Bi_32.5_S_67.5_ and XRD analysis indicated a Bi_2_S_3_ orthorhombic (ortho) crystal pattern exclusively. (ESI) HRTEM also showed lattice spacings corresponding to Bi_2_S_3_ ortho structure with fringes at 0.31 nm (211), 0.35 nm (130) and 0.51 nm (120) on the lateral face of nanoplates and 0.56 nm (020) along the axial profile of the nanoplates. (Fig. 3) UV-IR absorption spectroscopy showed a broad absorbance for these nanoplates across the visible and IR region, with a peak at 770 nm. (ESI) Visible absorbance for this material is associated with photoconductivity and thermoelectric properties^39,40^.

### Copper selenide

**Figure 4:**
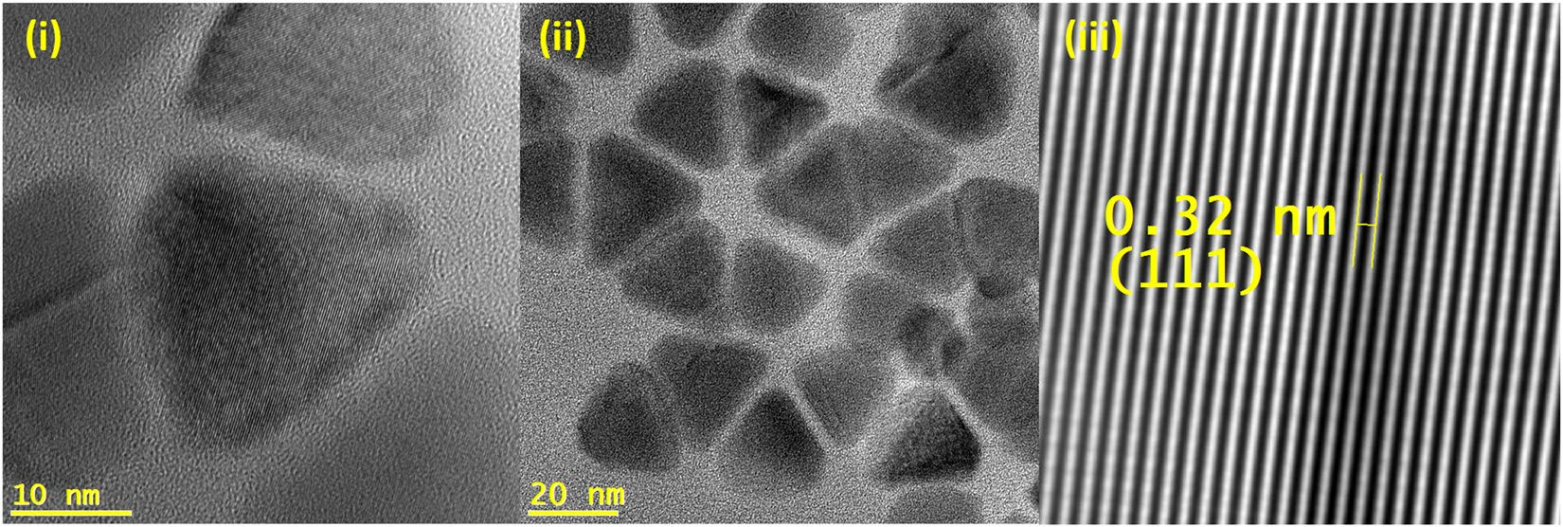
(**i)**,(**ii**) TEM images of copper selenide nanoplates, (**iii**) masked HRTEM of copper selenide nanoplates from lateral profile.

In this experiment, CuCl_2_.H_2_O was added to reduced selenium by hot injection. Unlike sulfur reduction, which occurs >80 °C, selenium reduction needs higher temperatures to fully reduced and subsequently dissolve (220 °C). Thus, in most cases, the reaction temperature was decreased before hot injection. In this case, the most effective reaction temperature was 180 °C for 30 min. Other temperatures showed non-uniform or mixed products, including nanoplates, nanowires and nanoparticles. For 180 °C, TEM showed small predominantly triangular nanoplates with a lateral length of 24.3 ± 3.4 nm and thickness of 8.6 ± 1.7 nm. EDX showed an elemental ratio of 65/35 for Cu/Se and XRD showed the pattern for Cu_1.8_Se (64/36) face centred cubic (fcc). (ESI) HRTEM showed the Cu_1.8_Se fcc (111) fringe exclusively from the lateral view. (Fig. 4) In optical studies, UV-IR absorption spectroscopy showed a broad NIR range peak and UV shoulder for this material. (ESI) The shoulder at 330 nm is attributed to the onset of band gap absorption for Cu_2−x_Se, while the broad peak at 1015 nm is due to plasmon absorption, and is attributed to the non-stoichiometry, which produces Cu vacancies, with the plasmon peak position a function of size, shape and doping level^41^.

### Iron selenide nanosheets

**Figure 5:**
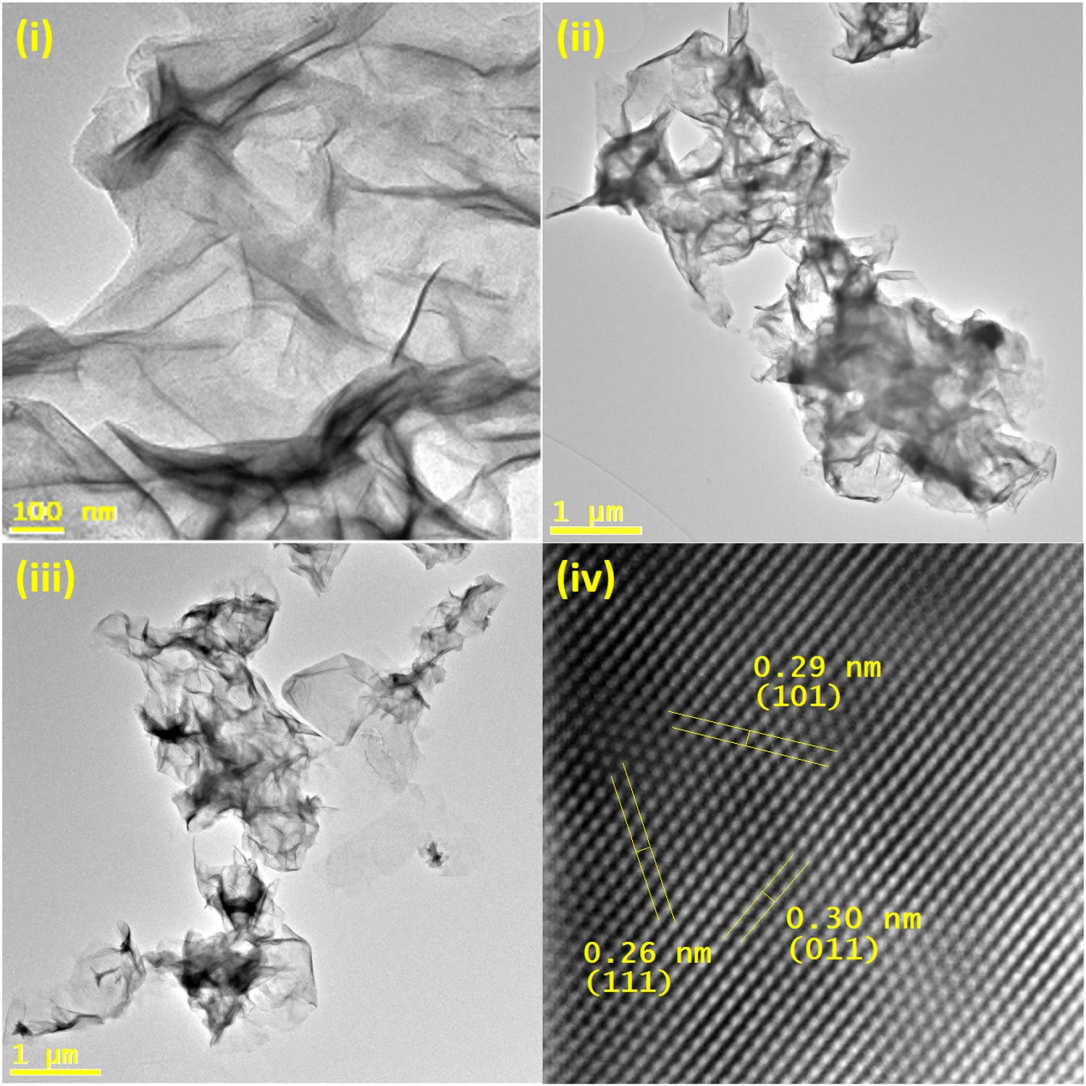
(**i)**–(**iii**) TEM images of iron selenide nanosheets, (**iv**) masked HRTEM from the lateral profile, (**iv**) masked HRTEM of FeSe_2_ ortho nanosheets.

This method used Fe(II)Cl_2_.H_2_O as the metal salt with reduced selenium. As with bismuth, iron is prone to oxidation, thus care must be taken in degassing and maintaining inert atmosphere, however in this case the metal salt dissolves freely in NMP. Optimum results were found at 160 °C, however the experiment can be successfully performed over the temperature range of 160–180 °C. For the product after 30 min at 160 °C, from TEM we can see that very thin sheets are formed with a lateral size in the micron to sub-micron range and <1 nm in thickness. The sheets are ruffled due to a drying effect, which can be seen by an increased density, produced by overlapping crystal planes **(**Fig. 5**)**.

EDX for this material showed a Fe/Se ratio of 30/70 and XRD showed a FeSe_2_ orthorhombic pattern for the nanosheets, indicating a selenium-rich surface. (ESI) HRTEM from the lateral view also confirmed the FeSe_2_ orthorhombic (ortho) structure with fringes present at 0.26 nm (111), 0.29 nm (101), 0.30 nm (011). **(**Fig. 5**)** Care must also be taken, during washing steps for this materials, as unreacted iron precursor can produce a by-product of small iron oxide nanoparticles, which is identifiable as a brown supernatant, whereas the iron selenide material is black. Prompt washing post-reaction and performing washing steps with CHCl_3_ (1% OAm) can inhibit this formation of iron oxide and remove the unreacted precursor. UV-IR absorption studies showed increasing absorbance from the visible into IR region, with a broad shoulder peak at 1170 nm. (ESI) Absorbance for this material is associated with photoconductivity^42,43^.

### Bismuth selenide nanosheets

**Figure 6:**
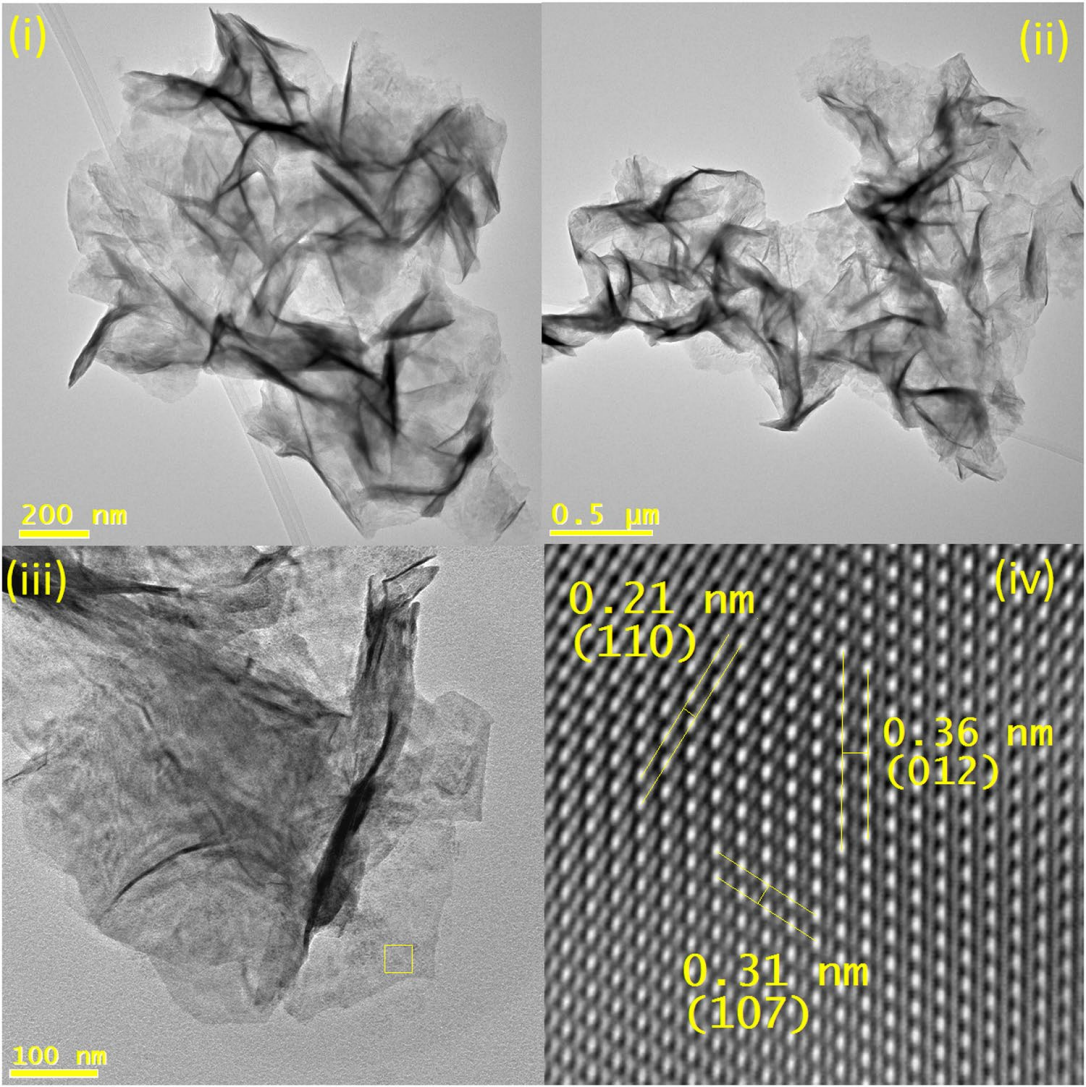
(**i**)–(**iii**) TEM images of Bi_4_Se_3_ rhombohedral nanosheets, (**iv**) masked HRTEM from the lateral profile.

Bismuth selenide nanostructures were produced using the Bi(III)Cl_3_ precursor dispersed in NMP and reduced selenium. After 30 min at 180 °C, nanosheets were formed as can be seen by TEM imaging. As with FeSe_2_ nanosheets, these were micron to sub-micron in lateral length and <1 nm in width, and also displayed a ruffling effect from drying. HRTEM showed lattice spacings for the Bi_4_Se_3_ rhom crystal structure. This structure is similar in nature to the well documented Bi_2_Se_3_ rhom crystal, and has similar properties^44^. (Fig. 6) EDX showed a ratio of Bi_55_Se_45_ close to the standard ratio of Bi_57_Se_43_ for this Bi_4_Se_3_ rhom structure (ESI).

XRD for the bismuth selenide nanosheets produced a diffraction pattern for the sandwiched sheets. Distinct diffraction points can be seen in this pattern, at regular intervals correlating to a distance of 0.735 nm. (ESI) This distance is due to the highly uniform thickness of the monolayer sheets. The distance between sheets is significantly larger due to surfactant separation (~2 nm) and is not present in the pattern^45^. UV-IR absorption studies showed a broad peak with a maximum at 540 nm. (ESI) Previous reports for few layer Bi_4_Se_3_ and closely related Bi_2_Se_3_ nanosheets, produced by exfoliation methods, have reported a broad peak in this region^44^. This peak is attributed to electron spin resonance. As a topological insulator, Bi_4_Se_3_ increases in metallic nature with decreasing layers. The monolayer material has optimal conductivity due to edge states, with applications in optoelectronics, among others.

## Conclusions

Thus a transferrable and adaptable synthetic route for a range of new anisotropic metal chalcogenide, nanomaterials has been developed here using an optimised, adaptable hot injection approach in surfactant solvent. A key advantage of this synthesis is its economy of reagents, which lends to scaling, cost-effectiveness and reproducibility of the reaction. Overall, this work has presented methods for the synthesis of anisotropic nanomaterials of different compositions and shapes, with excellent uniformity using this robust wet chemical approach.

## Materials and Methods

Reagents were used as purchased without further purification unless otherwise stated. We recommend purchase of smaller quantities of oxygen and water reactive reagents, to avoid oxidation and contamination over time, particularly for bismuth and iron salts. Detailed methods are contained in the ESI.

### General Metal Sulfide Synthesis

OAm and sulfur was degassed under vacuum at 80 °C for 30 min. The temperature was then raised to the reaction temperature under Ar, then rapid addition of the metal precursor, followed by stirring under Ar at the reaction temperature for varying amounts of time. Reactions were stopped by cooling the RBF in ice water, followed by methanol addition and centrifugation. Washings were performed by re-suspension with CHCl_3_ (1% OAm) and precipitation two times, followed by resuspension in CHCl_3_.

### General Metal Selenide Synthesis

OAm and selenium was degassed under vacuum at 80 °C for 30 min, then the temperature was raised to 220 °C under Ar until the Se(0) precursor was fully reduced to Se^2−^ and dissolved. The temperature was then reduced to the reaction temperature, then rapid addition of the metal precursor, followed by stirring under Ar at the reaction temperature for varying amounts of time. Reaction stopped by cooling the RBF with ice water, followed by methanol addition and centrifugation. Washings were performed by re-suspension with CHCl_3_ (1% OAm) and precipitation two times, followed by resuspension in CHCl_3_.

### Characterisation Methods

For TEM analysis, the materials were dispersed at approximately 0.1 mgmL^−1^ in hexane or chloroform by sonication for ~5 min in a sonic bath. They were then deposited on a 300 mesh thin carbon or holey thin carbon coated copper or molybdenum grids using a 1.5 µl aliquot of this dispersion. Copper containing samples were instead deposited on Mo grids, with the same coating, in order to measure elemental ratio by EDX. Measurements were performed using a Fei Titan microscope at an accelerating voltage of 400 kV, using bright field imaging. Size measurement were calculated from >100 measurements in the resulting images using ImageJ.

XRD was performed using a PANalytical X’Pert Powder diffractometer. The sample was prepared from a saturated solution in hexane or chloroform, then deposited onto a glass slide by pipette, with drying then additional depositions to build a dense layer of material with good adhesion to the glass. The XRD was measured over 3–6 hours for angles 10–155 (2θ). Baseline correction and smoothing were also performed.

ATR FTIR spectroscopy was performed using a Perkin Elmer Spectrum One NTS FTIR spectrometer. The liquid samples were deposited directly onto the diamond crystal. No further pressure was applied for crystal contact. ATR sampling was then performed over 10 min between 350–4000 cm^−1^. Baseline correction and smoothing were also performed.

Raman spectra were registered in backscattering geometry using a Renishaw 1000 micro-Raman system, equipped with Leica microscopes. To prevent sample heating, the laser power was kept below 3 mW on sample. Measurements were performed at room temperature with a He-Ne laser at 633 nm excitation. Long working distance x50 Olympus microscope objective was used to focus the laser light onto sample with a spot diameter of ~4 μm. An 1800 lines/mm grating was used for all measurements, giving a spectral resolution of ~1 cm^−1^. All spectra were registered in the extended mode in the spectral range from 150 to 2000 cm^−1^ with the same accumulation time of 100 s. Baseline correction and smoothing were also performed.

UV-IR absorption measurements were performed using a Perkin Elmer Lambda1050 spectrometer. Samples were prepared by dispersion in hexane or chloroform and measurements were run over 250–2500 nm, at 2 nm intervals. In some cases smoothing was performed.

### Availability of data and materials

The datasets and materials generated during and/or analysed during the current study are available from the corresponding author upon reasonable request.

## Electronic supplementary material

Supplementary Information
