# Supplementary material for: Adaptable surfactant-mediated method for the preparation of anisotropic metal chalcogenide nanomaterials
Source: Sci Rep. 2018 Feb 12;8:2860. doi: 10.1038/s41598-018-21328-7 (PMC5809463; doi:10.1038/s41598-018-21328-7)

Adaptable surfactant-mediated method for the preparation of anisotropic metal chalcogenide nanomaterials

S. A. McCarthy[1,2], R. Ratkic[1], F. Purcell-Milton[1,2], Tatiana S. Perova[3], Y. K. Gun'ko[1,2,4]

[1] School of Chemistry, Trinity College Dublin, Dublin 2, Ireland

[2] CRANN, Trinity College Dublin, Dublin 2, Ireland

[3] Department of Electronic and Electrical Engineering

[4] ITMO University, St. Petersburg, Russia

SUPPORTING INFORMATION

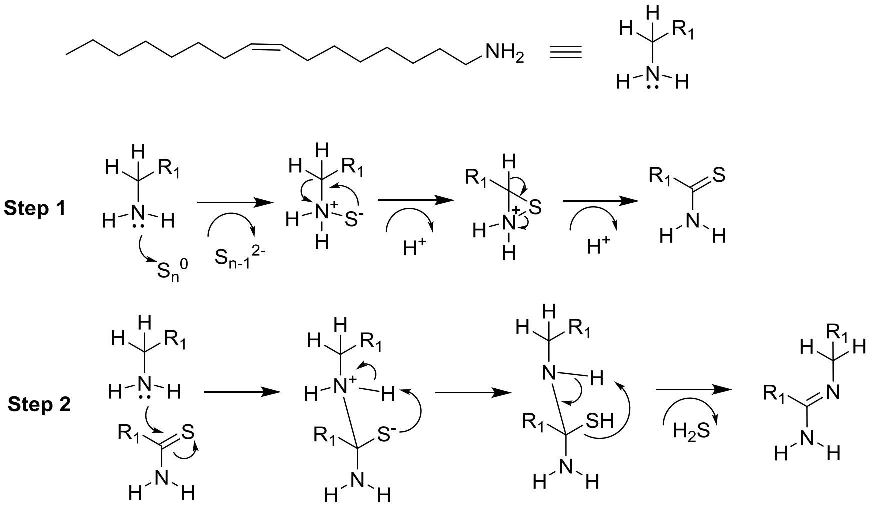

Figure: Proposed mechanism for reduction of elemental sulfur to S^{2-} by OAm through a two-amine reduction to produce a branched amidine

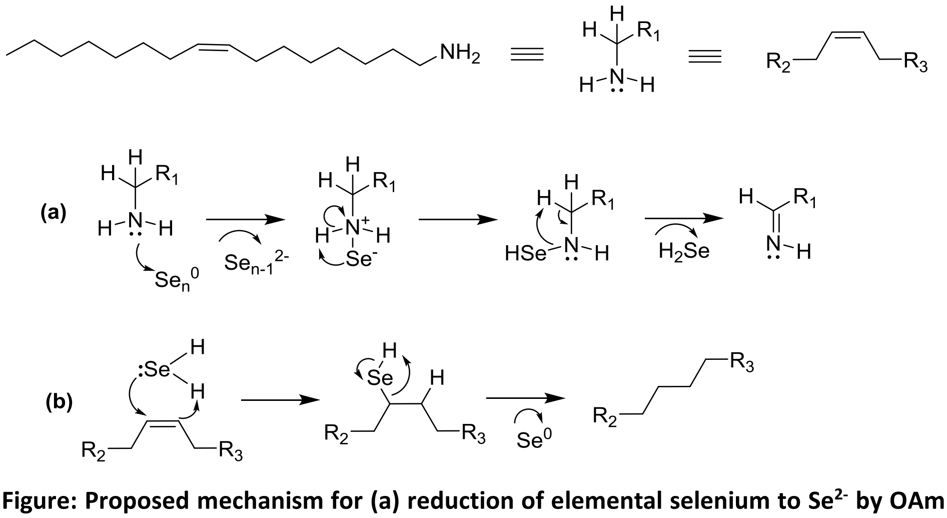

Figure: Proposed mechanism for (a) reduction of elemental selenium to Se^{2-} by OAm and (b) side reaction of olefin reduction by hydrogen selenide

FTIR and Raman spectroscopic data for reduction of S/Se by OAm

Abbreviations from the graphs: (Str.) stretching vibration **(Bend.)** bending vibration, **(Asym.)** asymmetric, **(Sym.)** symmetric, **(Def.)** deformation vibration, **(Twist.)** twisting vibration, **(Rock.)** rocking vibration

---

FTIR and Raman graphs for oleylamine-sulfur reaction

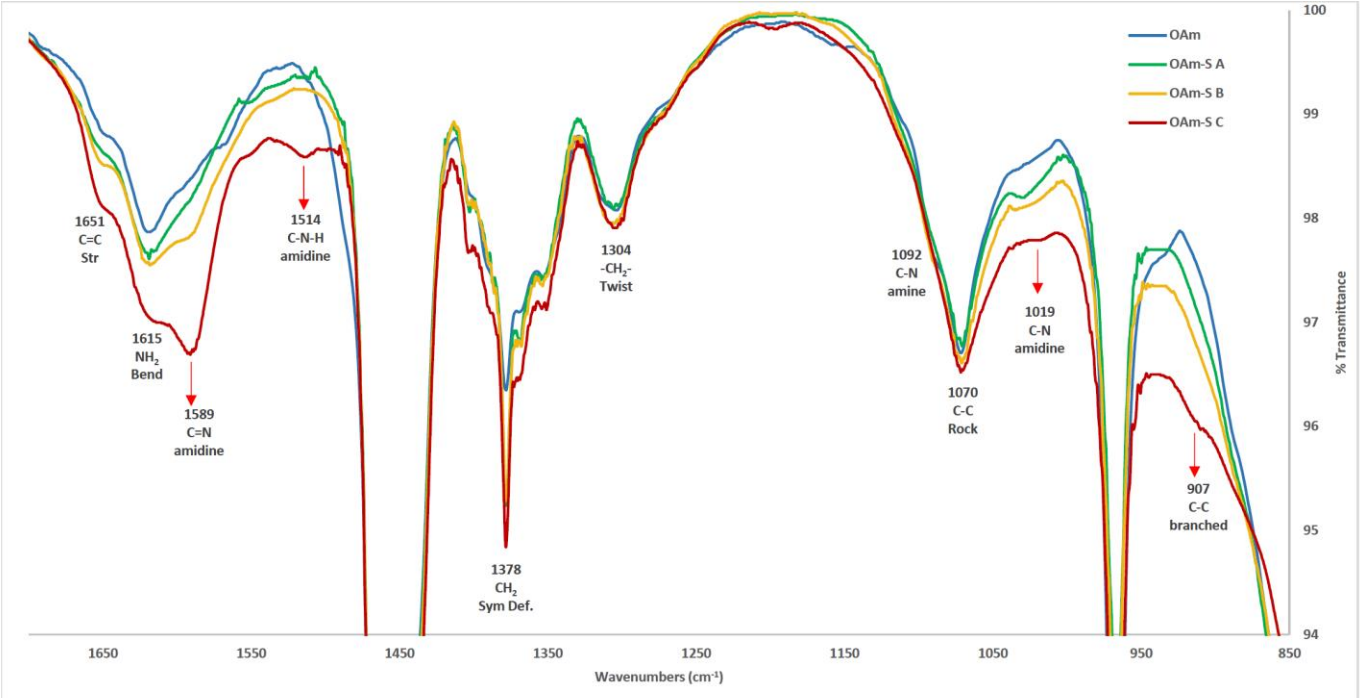

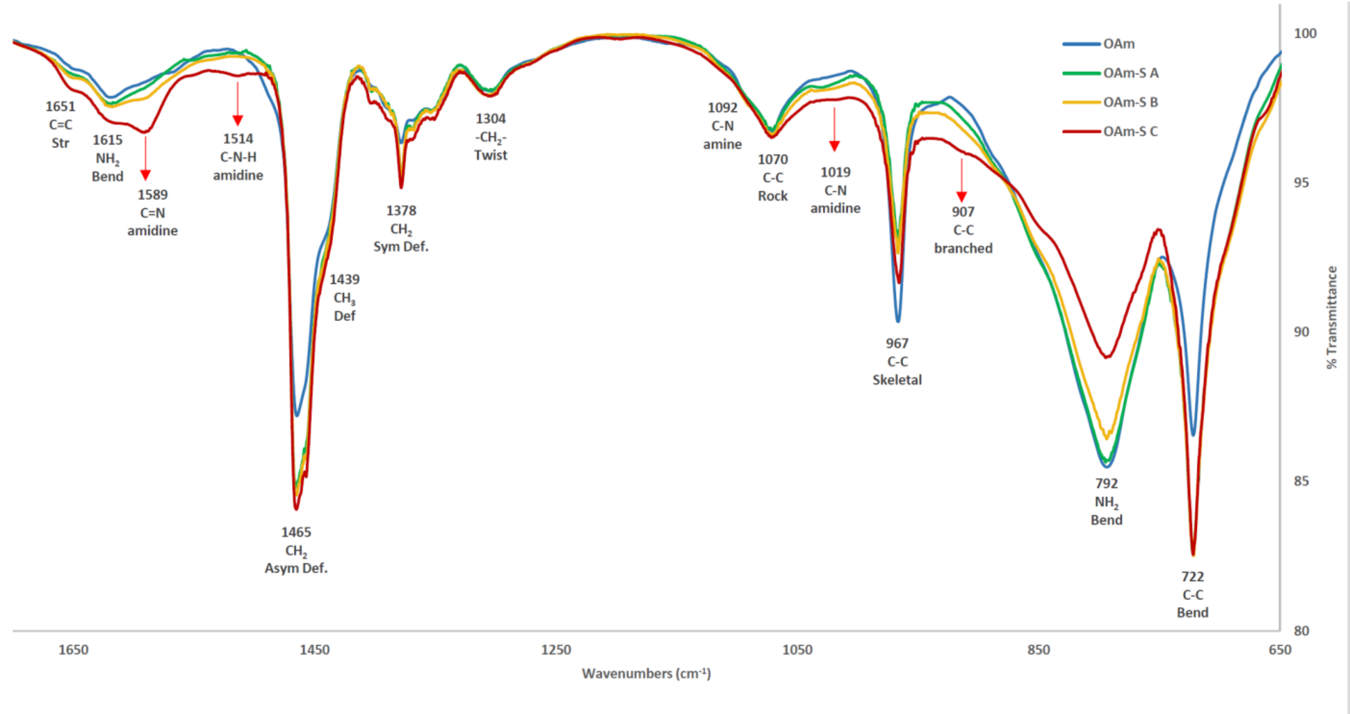

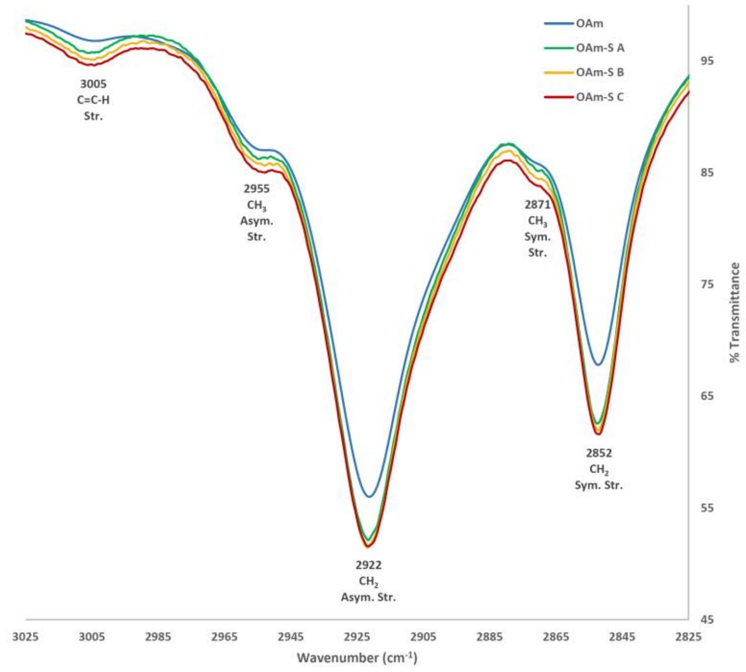
OAm
OAm-S A
OAm-S B
OAm-S C
3005
C=C-H
Str.
2955
CH3
Asym.
Str.
2871
CH3
Sym.
Str.
2852
CH2
Sym. Str.
2922
CH2
Asym. Str.
95
85
75
65
55
45
% Transmittance
3025
3005
2985
2965
2945
2925
2905
2885
2865
2845
2825
Wavenumber (cm-1)

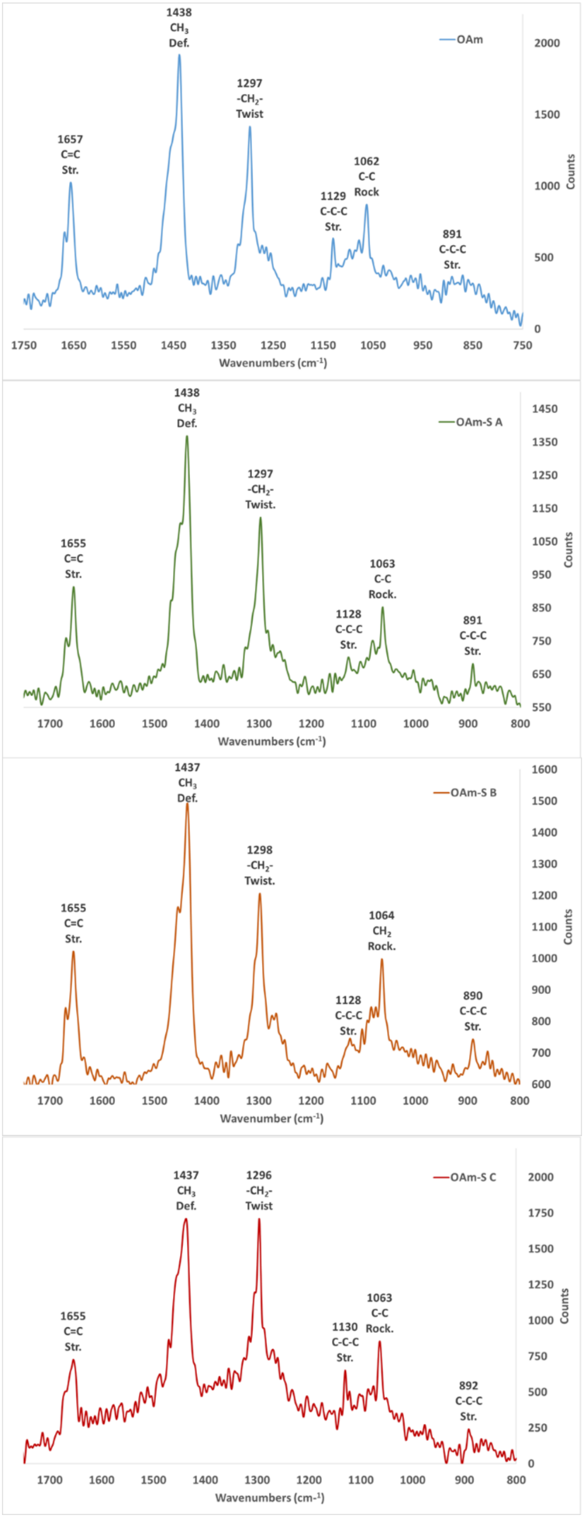
OAm
1438
CH3
Def.
1297
-CH2-
Twist
1657
C=C
Str.
1062
C-C
Rock
1129
C-C-C
Str.
891
C-C-C
Str.
Counts
Wavenumbers (cm-1)
OAm-S A
1438
CH3
Def.
1297
-CH2-
Twist.
1655
C=C
Str.
1063
C-C
Rock.
1128
C-C-C
Str.
891
C-C-C
Str.
Counts
Wavenumbers (cm-1)
OAm-S B
1437
CH3
Def.
1298
-CH2-
Twist.
1655
C=C
Str.
1064
CH2
Rock.
1128
C-C-C
Str.
890
C-C-C
Str.
Counts
Wavenumber (cm-1)
OAm-S C
1437
CH3
Def.
1296
-CH2-
Twist
1655
C=C
Str.
1063
C-C
Rock.
1130
C-C-C
Str.
892
C-C-C
Str.
Counts
Wavenumbers (cm-1)

FTIR and Raman graphs for oleylamine-selenium reaction

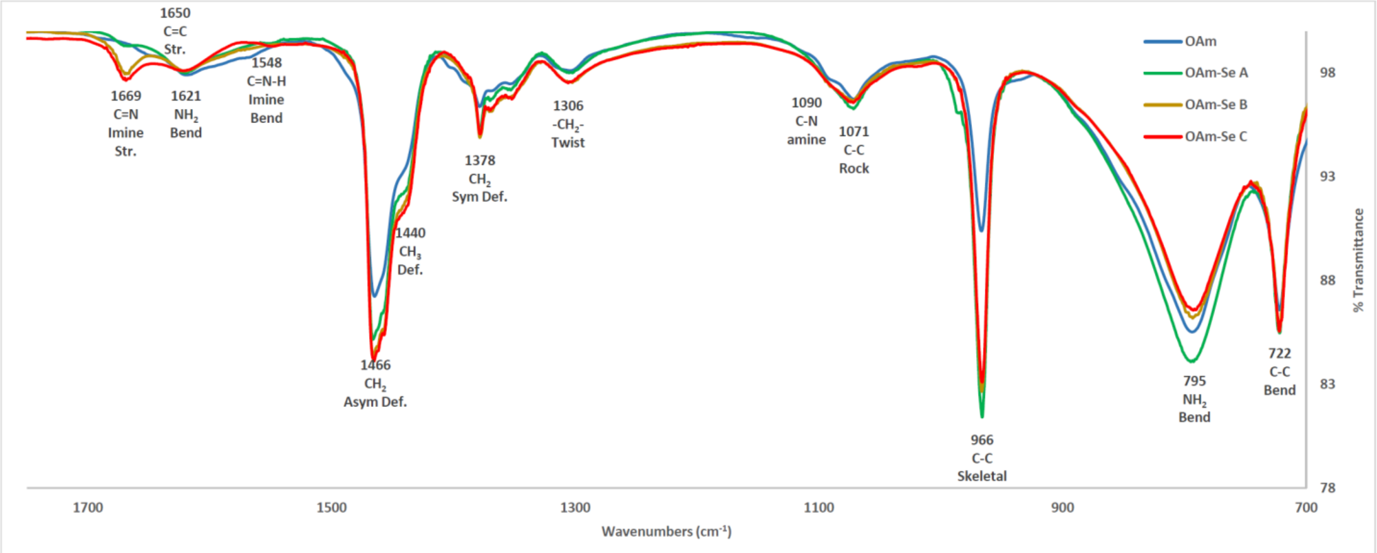

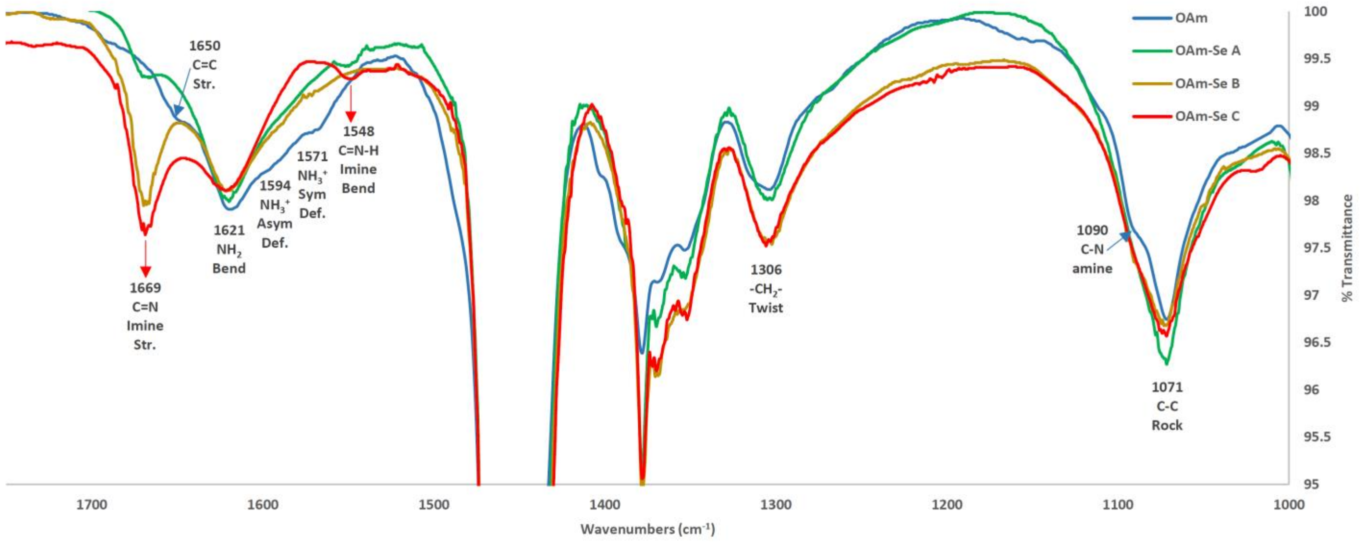

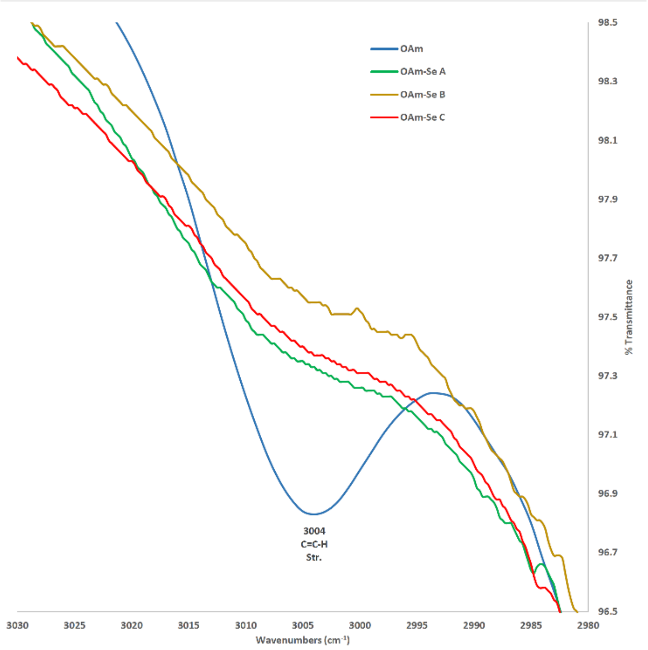
OAm
OAm-Se A
OAm-Se B
OAm-Se C
3004
C=C-H
Str.
% Transmittance
Wavenumbers (cm⁻¹)

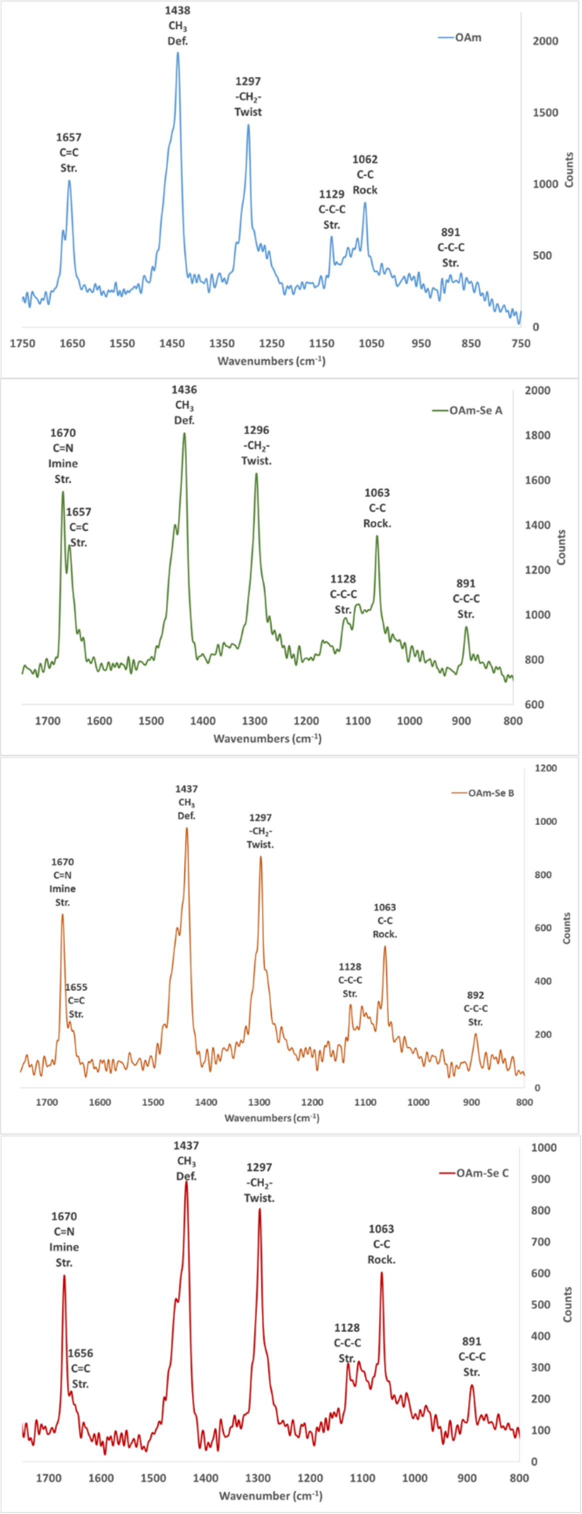
OAm
1438
CH3
Def.
1297
-CH2-
Twist
1657
C=C
Str.
1062
C-C
Rock
1129
C-C-C
Str.
891
C-C-C
Str.
Counts
Wavenumbers (cm⁻¹)
OAm-Se A
1436
CH3
Def.
1296
-CH2-
Twist.
1670
C=N
Imine
Str.
1657
C=C
Str.
1063
C-C
Rock.
1128
C-C-C
Str.
891
C-C-C
Str.
OAm-Se B
1437
CH3
Def.
1297
-CH2-
Twist.
1670
C=N
Imine
Str.
1655
C=C
Str.
1063
C-C
Rock.
1128
C-C-C
Str.
892
C-C-C
Str.
OAm-Se C
1437
CH3
Def.
1297
-CH2-
Twist.
1670
C=N
Imine
Str.
1656
C=C
Str.
1063
C-C
Rock.
1128
C-C-C
Str.
891
C-C-C
Str.
Wavenumber (cm⁻¹)

METHODS

General Metal Sulfide Synthesis:

A mixture of OAm (50 mL) and sulfur was degassed under vacuum at 80°C for 30 min. The temperature was then raised to the reaction temperature under Ar, then rapid addition of the metal precursor, followed by stirring under Ar at the reaction temperature for varying amounts of time. Reactions were stopped by cooling the RBF under tap water, followed by methanol addition and centrifugation. Washings were performed by re-suspension with $CHCl_3$ (1% OAm) and precipitation two times, followed by resuspension in $CHCl_3$.

General Metal Selenide Synthesis:

A mixture of OAm (50 mL) and selenium was degassed under vacuum at 80°C for 30 min, then the temperature was raised to 220^{0}C under Ar until the Se(0) precursor was fully reduced to Se^{2-} and dissolved. The temperature was then reduced to the reaction temperature, then rapid addition of the metal precursor, followed by stirring under Ar at the reaction temperature for varying amounts of time. Reaction stopped by cooling the RBF under tap water, followed by methanol addition and centrifugation. Washings were performed by re-suspension with $CHCl_3$ (1% OAm) and precipitation two times, followed by resuspension in $CHCl_3$.

Table S1: Summary of Reaction Conditions and Results

Material	Chalcogen (Ch) precursor	Ch mmol	Metal (M) Precursor	M mmol	Time (min)	Temp. (ºC)	Conc. (mM) (M/Ch)	EDX Ratio (M/Ch)	Crystal	Shape	Size (nm) Lateral|Axial	Absorption (nm)
CuS	S	2	$CuCl_2.H_2O$	1	30	120	20/40	52/48	CuS hex	Nanoplate	55.5| 4.15	400, 1200
CuS	S	3	$CuCl_2.H_2O$	1	30	180	20/60	55/45	CuS hex	Nanoplate	755 | ≤1	420, IR
Bi_2S_3	S	3	$BiCl_3$	1	30	180	20/0	32.5/67.5	Bi_2S_3 ortho	Nanoplate	67.5 | 10.5	770
$Cu_{1.8}Se$	Se	2	$CuCl_2.H_2O$	1	30	180	20/40	65/35	$Cu_{1.8}Se$ fcc	Nanoplate	24.3 | 8.6	1015
$FeSe_2$	Se	3	$FeCl_2.4H_2O$	1	30	160	20/40	30/70	$FeSe_2$ ortho	Nanosheet	>1000 | <1	1170, IR
Bi_4Se_3	Se	3	$BiCl_3$	1	30	180	20/60	55.5/44.5	Bi_4Se_3 rhom	Nanosheet	>1000 | <1	540

Copper Sulfide I Synthesis

OAm (50 mL) was added to a RBF (100 mL) attached to a Schlenk line. The reaction was put under vacuum and the temperature was raised to 80^{0}C. After 30min at 80^{0}C the reaction was put under Ar, and then elemental sulfur (2 mmol) was added to the reaction, under an Ar flow to avoid oxygen introduction into the RBF. The temperature was then raised to 120^{0}C. A solution was prepared of $CuCl_2.H_2O$ (1 mmol) in NMP (5 mL). The copper solution was added to the reaction rapidly by syringe under an Ar flow. The reaction was then stirred for 30 mins at 120^{0}C. The reaction was stopped by cooling the RBF in an ice bath followed by addition of MeOH (50 mL). The mixture was agitated briefly using a vortex, then separated into 4 centrifuge tubes for centrifugation to remove excess OAm and salts. The precipitated product was collected and the supernatant carefully removed and discarded. The product was then recombined into one centrifuge tube with a solution of OAm in $CHCl_3$ (1%OAm, 20 mL). The dispersion was then mixed by vortex and then precipitated again by centrifugation. Washing with $CHCl_3$ (1 % OAm) was repeated, then the precipitate was then dispersed in $CHCl_3$ (20 mL) to yield the final product.

Copper Sulfide II Synthesis

OAm (50 mL) was added to a RBF (100 mL) attached to a Schlenk line. The reaction was put under vacuum and the temperature was raised to 80^{0}C. After 30min at 80^{0}C the reaction was put under Ar, and then elemental sulfur (3 mmol) was added to the reaction, under an Ar flow to avoid oxygen introduction into the RBF. The temperature was then raised to 180^{0}C. A solution was prepared of $CuCl_2.H_2O$ (1 mmol) in NMP (5 mL). The copper solution was added to the reaction rapidly by syringe under an Ar flow. The reaction was then stirred for 30 mins at 180^{0}C. The reaction was stopped by

cooling the RBF in an ice bath followed by addition of MeOH (50 mL). The mixture was agitated briefly using a vortex, then separated into 4 centrifuge tubes for centrifugation to remove excess OAm and salts. The precipitated product was collected and the supernatant carefully removed and discarded. The product was then recombined into one centrifuge tube with a solution of OAm in $CHCl_3$ (1%OAm, 20 mL). The dispersion was then mixed by vortex and then precipitated again by centrifugation. Washing with $CHCl_3$ (1 % OAm) was repeated, then the precipitate was then dispersed in $CHCl_3$ (20 mL) to yield the final product.

Bismuth Sulfide Synthesis

OAm (50 mL) was added to a RBF (100 mL) attached to a Schlenk line. The reaction was put under vacuum and the temperature was raised to 80^0C. After 30min at 80^0C the reaction was put under Ar, and then elemental sulfur (3 mmol) was added to the reaction, under an Ar flow to avoid oxygen introduction into the RBF. The temperature was then raised to 180^0C. A dispersion was prepared of $BiCl_3$ (1 mmol) in NMP (5 mL). Note, this salt does not dissolve in NMP but forms a fine dispersion after sonication for 5 min. The bismuth dispersion was added to the reaction rapidly by syringe under an Ar flow. The reaction was then stirred for 30 mins at 180^0C. The reaction was stopped by cooling the RBF in an ice bath followed by addition of MeOH (50 mL). The mixture was agitated briefly using a vortex, then separated into 4 centrifuge tubes for centrifugation to remove excess OAm and salts. The precipitated product was collected and the supernatant carefully removed and discarded. The product was then recombined into one centrifuge tube with a solution of OAm in $CHCl_3$ (1%OAm, 20 mL). The dispersion was then mixed by vortex and then precipitated again by centrifugation. Washing with $CHCl_3$ (1 % OAm) was repeated, then the precipitate was then dispersed in $CHCl_3$ (20 mL) to yield the final product.

Copper Selenide Synthesis

OAm (50 mL) was added to a RBF (100 mL) attached to a Schlenk line. The reaction was put under vacuum and the temperature was raised to 80^0C. After 30min at 80^0C the reaction was put under Ar, and then elemental selenium (2 mmol) was added to the reaction, under an Ar flow to avoid oxygen introduction into the RBF. The temperature was then raised to 220^0C. The reaction was stirred at 220^0C until the Se was fully dissolved and reduced, as indication by the disappearance of black solid in the RBF. The temperature was then reduced to 180^0C.

A solution was prepared of $CuCl_2.H_2O$ (1 mmol) in NMP (5 mL). The copper solution was added to the reaction rapidly by syringe under an Ar flow. The reaction was then stirred for 30 mins at 180^0C. The reaction was stopped by cooling the RBF in an ice bath followed by addition of MeOH (50 mL). The mixture was agitated briefly using a vortex, then separated into 4 centrifuge tubes for centrifugation to remove excess OAm and salts. The precipitated product was collected and the supernatant carefully removed and discarded. The product was then recombined into one centrifuge tube with a solution of OAm in $CHCl_3$ (1%OAm, 20 mL). The dispersion was then mixed by vortex and then precipitated again by centrifugation. Washing with $CHCl_3$ (1 % OAm) was repeated, then the precipitate was then dispersed in $CHCl_3$ (20 mL) to yield the final product.

Iron Selenide Synthesis

OAm (50 mL) was added to a RBF (100 mL) attached to a Schlenk line. The reaction was put under vacuum and the temperature was raised to 80^{0}C. After 30min at 80^{0}C the reaction was put under Ar, and then elemental selenium (3 mmol) was added to the reaction, under an Ar flow to avoid oxygen introduction into the RBF. The temperature was then raised to 220^{0}C. The reaction was stirred at 220^{0}C until the Se was fully dissolved and reduced, as indication by the disappearance of black solid in the RBF. The temperature was then reduced to 160^{0}C

A solution was prepared of $FeCl_2.4H_2O$ (1 mmol) in NMP (5 mL). The copper solution was added to the reaction rapidly by syringe under an Ar flow. The reaction was then stirred for 30 mins at 160^{0}C. The reaction was stopped by cooling the RBF in an ice bath followed by addition of MeOH (50 mL). The mixture was agitated briefly using a vortex, then separated into 4 centrifuge tubes for centrifugation to remove excess OAm and salts. The precipitated product was collected and the supernatant carefully removed and discarded. The product was then recombined into one centrifuge tube with a solution of OAm in $CHCl_3$ (1%OAm, 20 mL). The dispersion was then mixed by vortex and then precipitated again by centrifugation. Washing with $CHCl_3$ (1 % OAm) was repeated, then the precipitate was then dispersed in $CHCl_3$ (20 mL) to yield the final product.

Bismuth Selenide Synthesis

OAm (50 mL) was added to a RBF (100 mL) attached to a Schlenk line. The reaction was put under vacuum and the temperature was raised to 80^{0}C. After 30min at 80^{0}C the reaction was put under Ar, and then elemental selenium (3 mmol) was added to the reaction, under an Ar flow to avoid oxygen introduction into the RBF. The temperature was then raised to 220^{0}C. The reaction was stirred at 220^{0}C until the Se was fully dissolved and reduced, as indication by the disappearance of black solid in the RBF. The temperature was then reduced to 180^{0}C

A dispersion was prepared of $BiCl_3$ (1 mmol) in NMP (5 mL). Note, this salt does not dissolve in NMP but forms a fine dispersion after sonication for 5 min. The bismuth dispersion was added to the reaction rapidly by syringe under an Ar flow. The reaction was then stirred for 30 mins at 180^{0}C. The reaction was stopped by cooling the RBF in an ice bath followed by addition of MeOH (50 mL). The mixture was agitated briefly using a vortex, then separated into 4 centrifuge tubes for centrifugation to remove excess OAm and salts. The precipitated product was collected and the supernatant carefully removed and discarded. The product was then recombined into one centrifuge tube with a solution of OAm in $CHCl_3$ (1%OAm, 20 mL). The dispersion was then mixed by vortex and then precipitated again by centrifugation. Washing with $CHCl_3$ (1 % OAm) was repeated, then the precipitate was then dispersed in $CHCl_3$ (20 mL) to yield the final product.

COPPER SULFIDE I

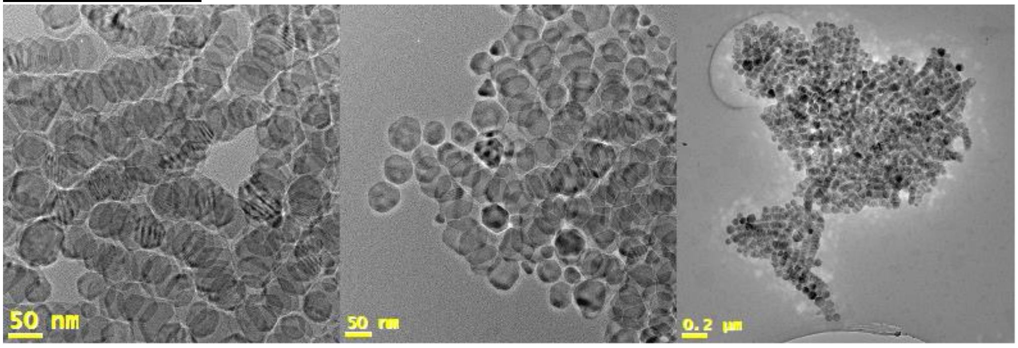

Figure: TEM images of small copper sulfide nanoplates

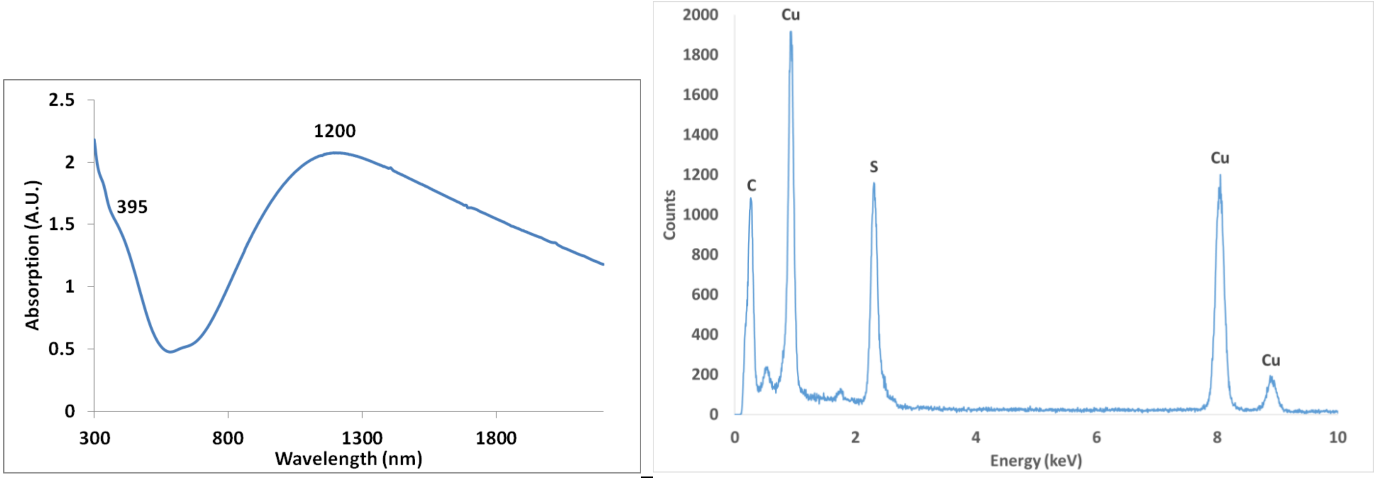

Figure: UV-IR absorption spectrum for small CuS nanoplates in chloroform (left), EDX spectrum of small CuS nanoplates showing a Cu/S ratio of 52/48 (right)

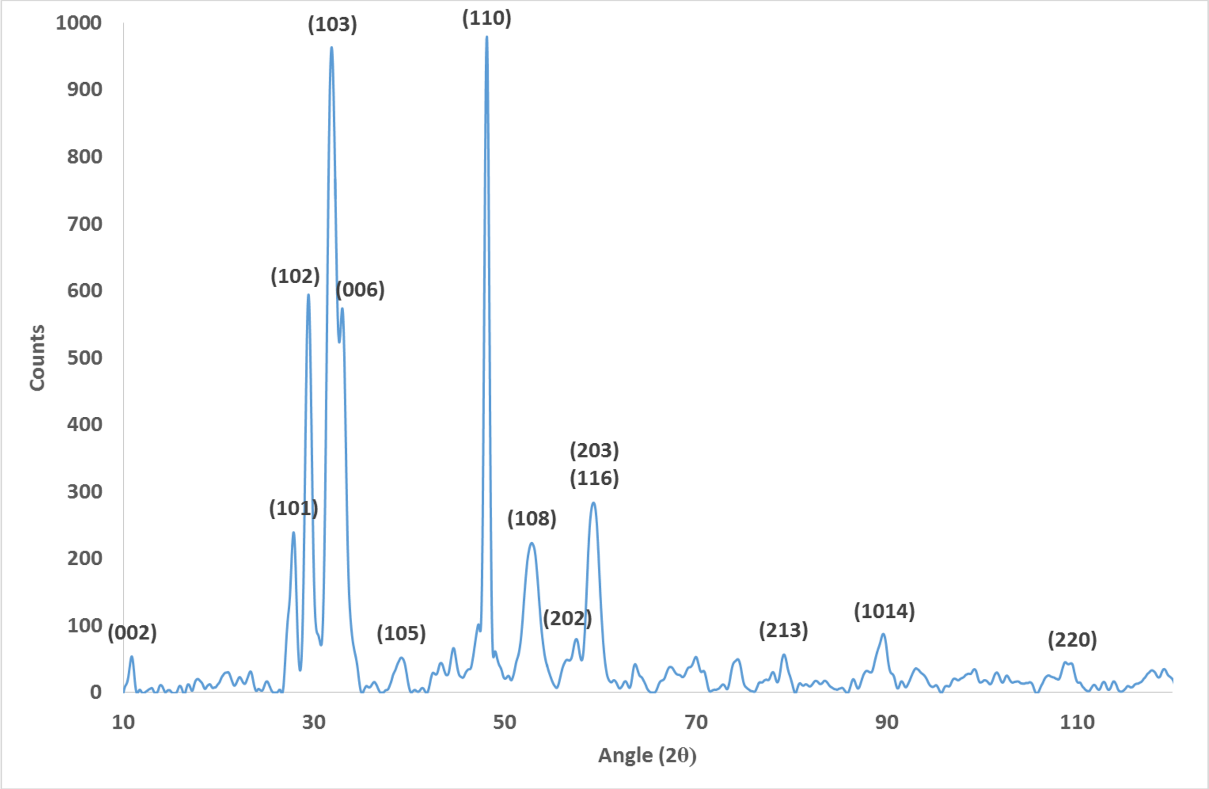

Figure: XRD pattern for small CuS hexagonal nanoplates

COPPER SULFIDE II

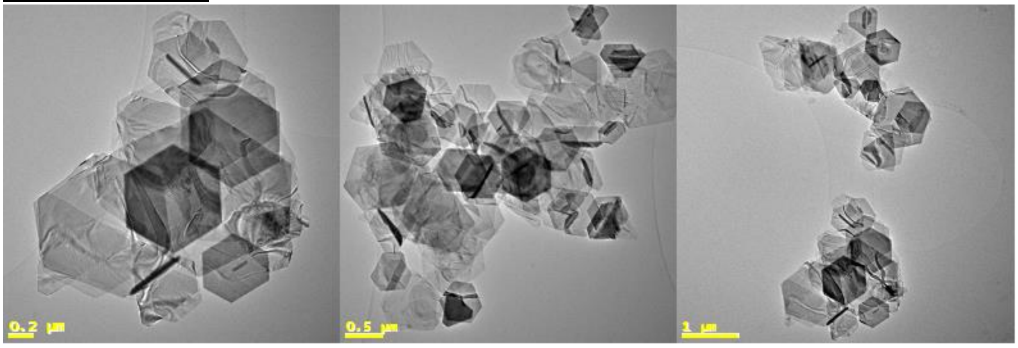

Figure: TEM images of large copper sulfide nanoplates

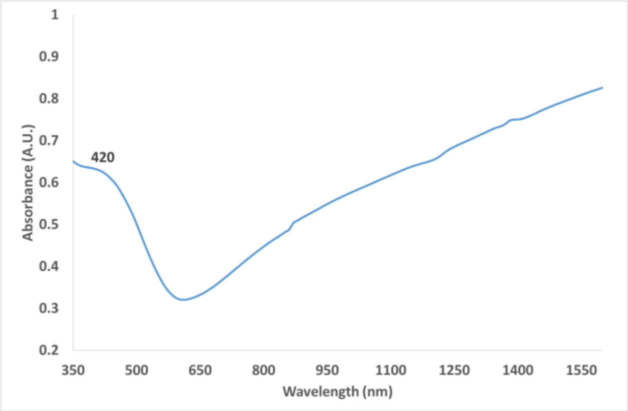

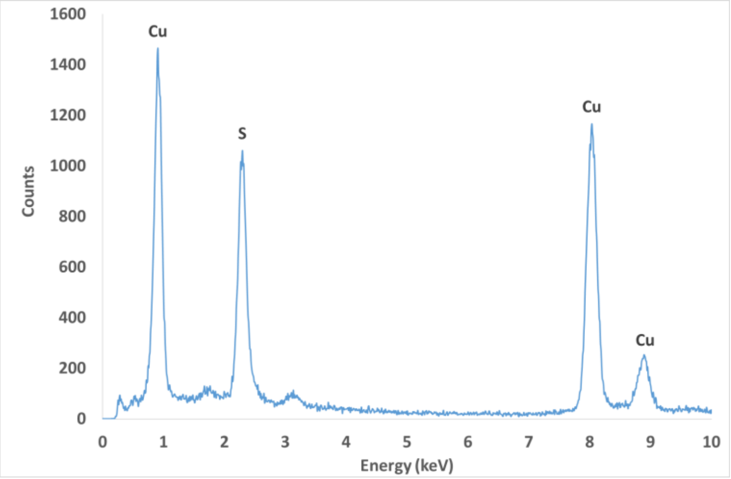

Figure: UV-IR absorption spectrum for large CuS nanoplates in chloroform (left), EDX spectrum of large CuS nanoplates showing a Cu/S ratio of 55/45 (right)

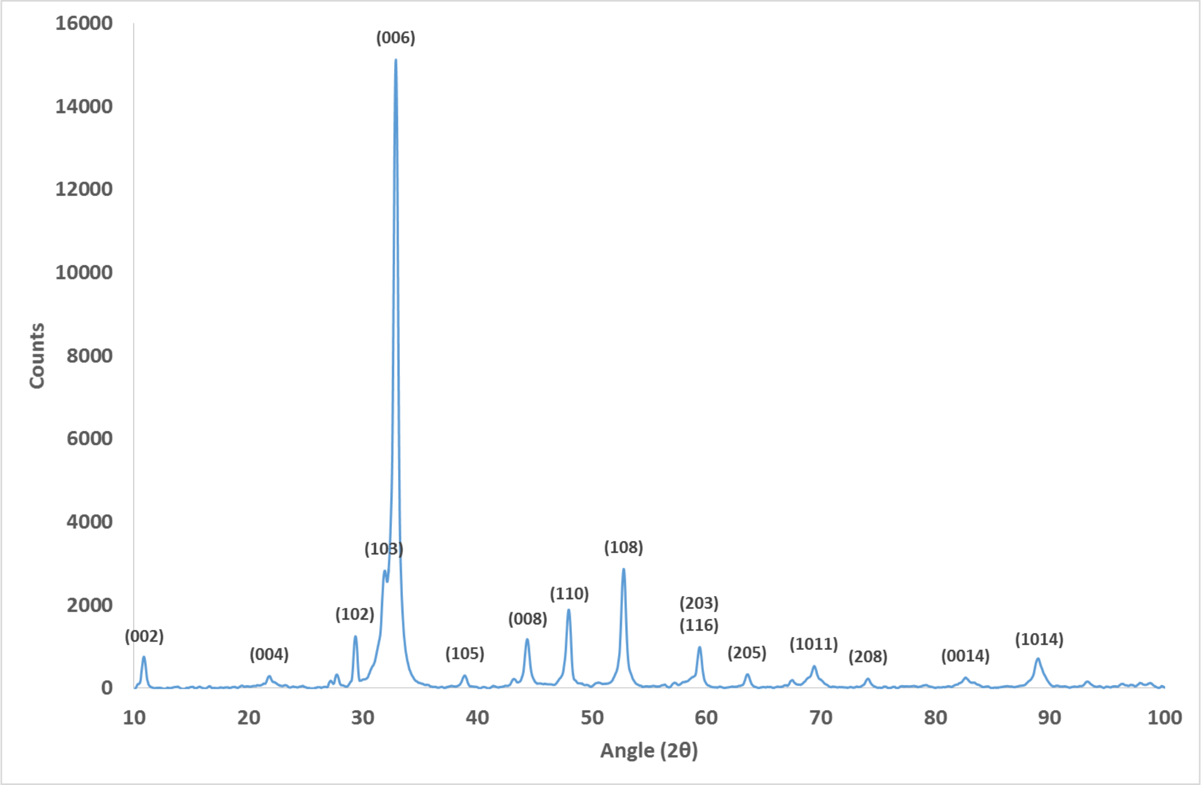

Figure: XRD pattern for CuS hexagonal large nanoplates

BISMUTH SULFIDE NANOSTRUCTURES

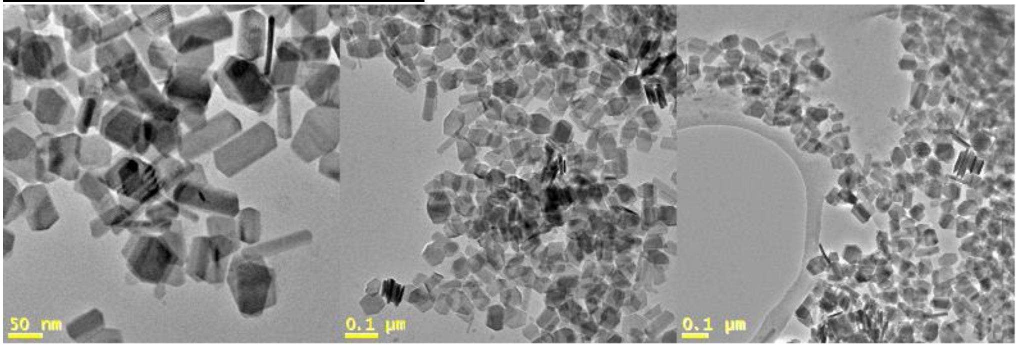

Figure: TEM images of Bi_2S_3 nanoplates

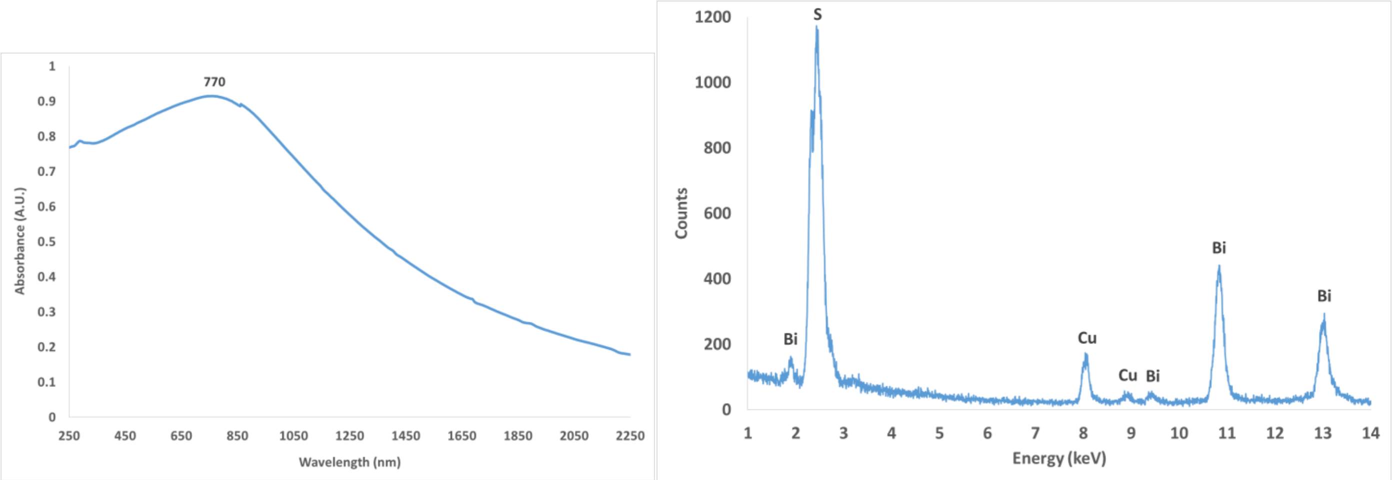

Figure: UV-IR absorption spectrum of Bi_2S_3 nanoplates in chloroform (left) EDX spectrum of Bi_2S_3 nanoplates showing a Bi/S ratio of 32.5/67.5 (right)

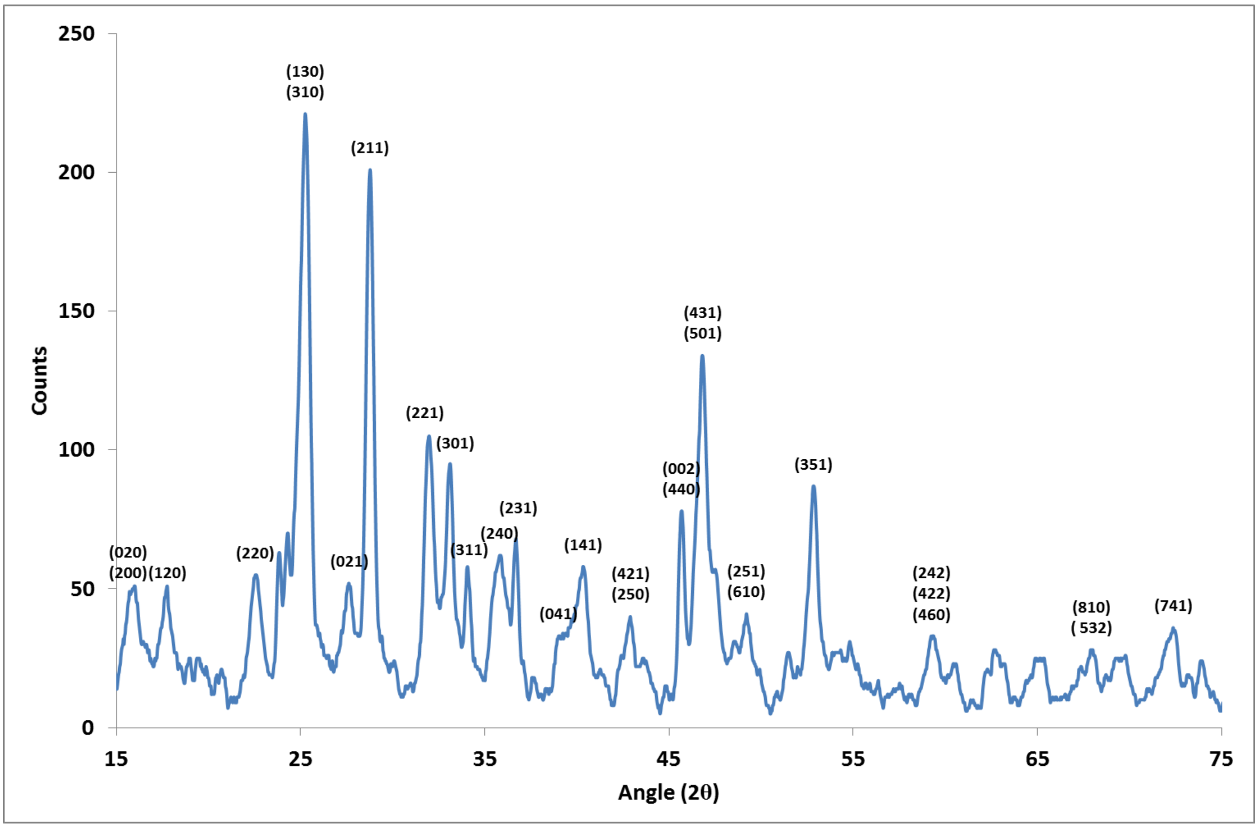

Figure: XRD pattern for Bi_2S_3 orthorhombic nanoplates

COPPER SELENIDE

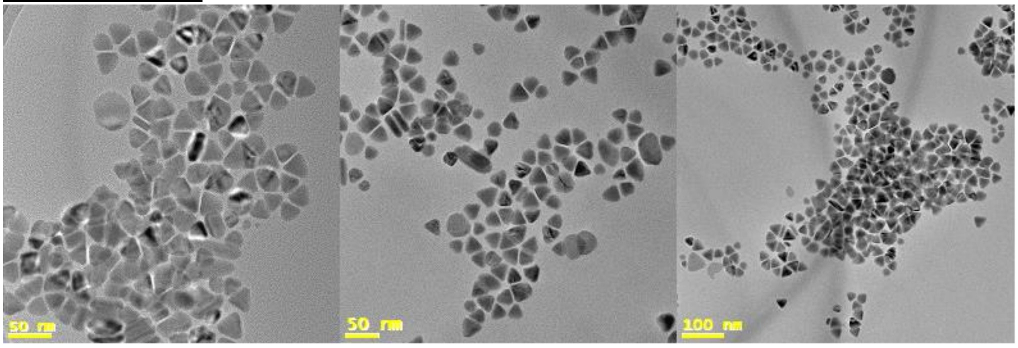

Figure: TEM images of copper selenide nanoplates

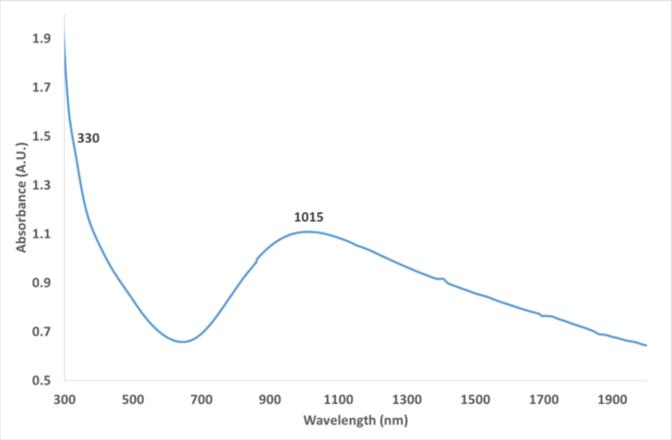

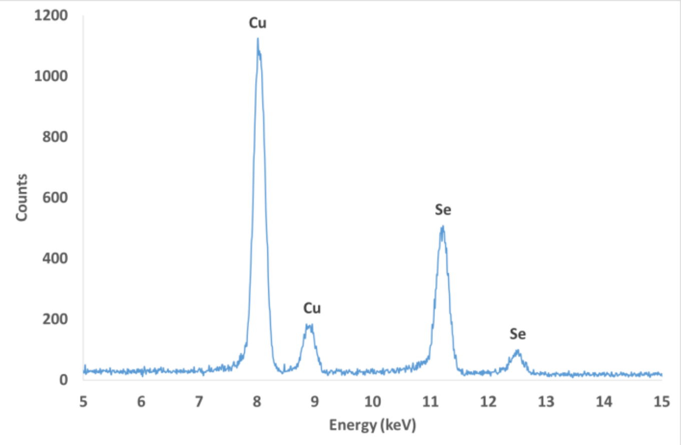

Figure: UV-IR absorption spectrum of copper selenide nanoplates in chloroform (left), EDX spectrum of $Cu_{1.8}Se$ nanoplates showing a Cu/Se ratio of 65/35 (right)

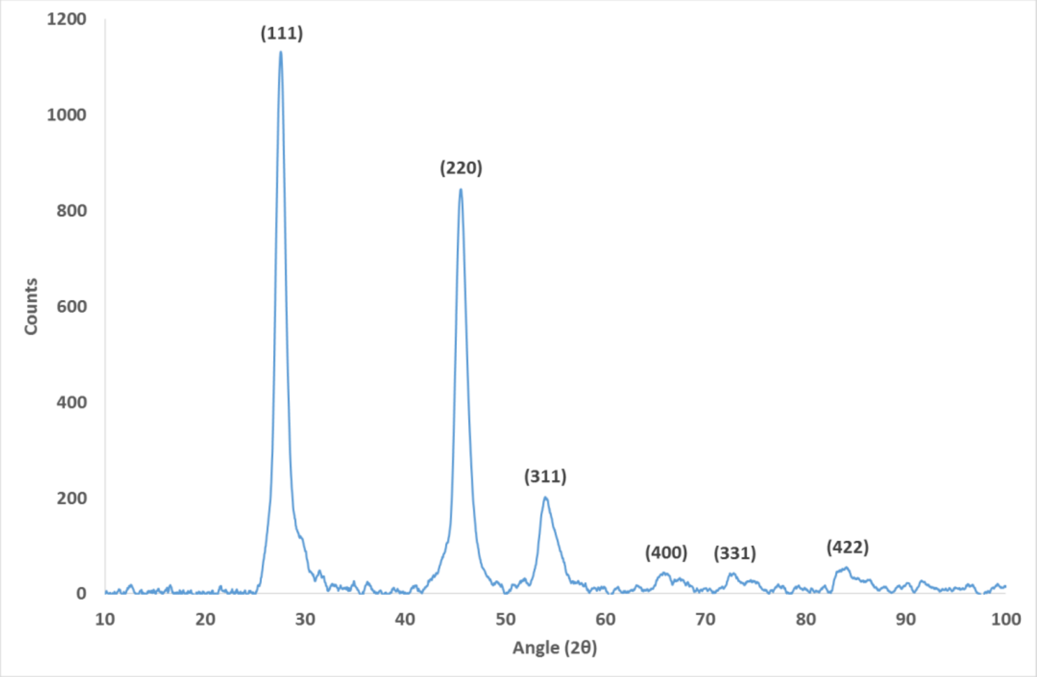

Figure: XRD pattern for $Cu_{1.8}Se$ face centred cubic nanoplates

IRON SELENIDE

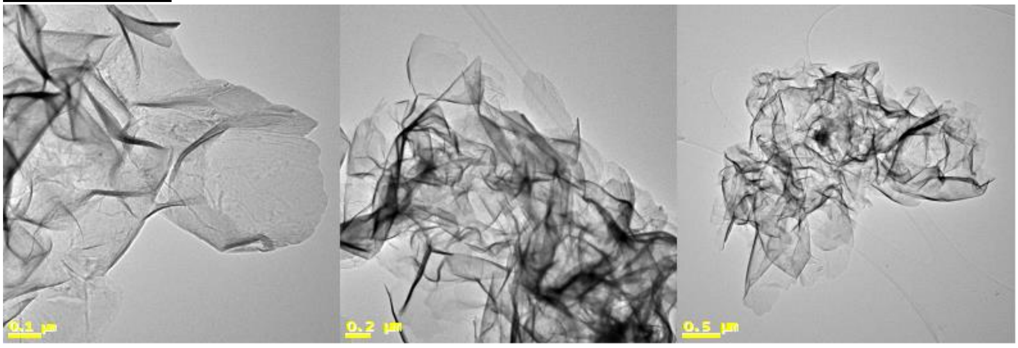

Figure: TEM images of $FeSe_2$ nanosheets

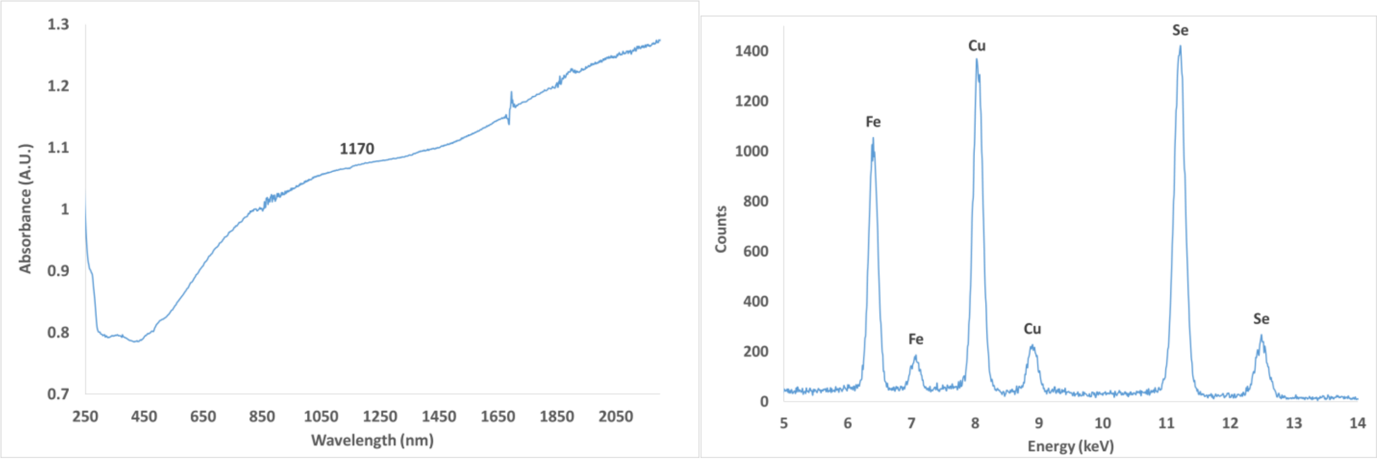

Figure: UV-IR absorption spectrum for $FeSe_2$ nanosheets in chloroform (left), EDX spectrum of $FeSe_2$ nanosheets showing a Fe/Se ratio of 30/70 (right)

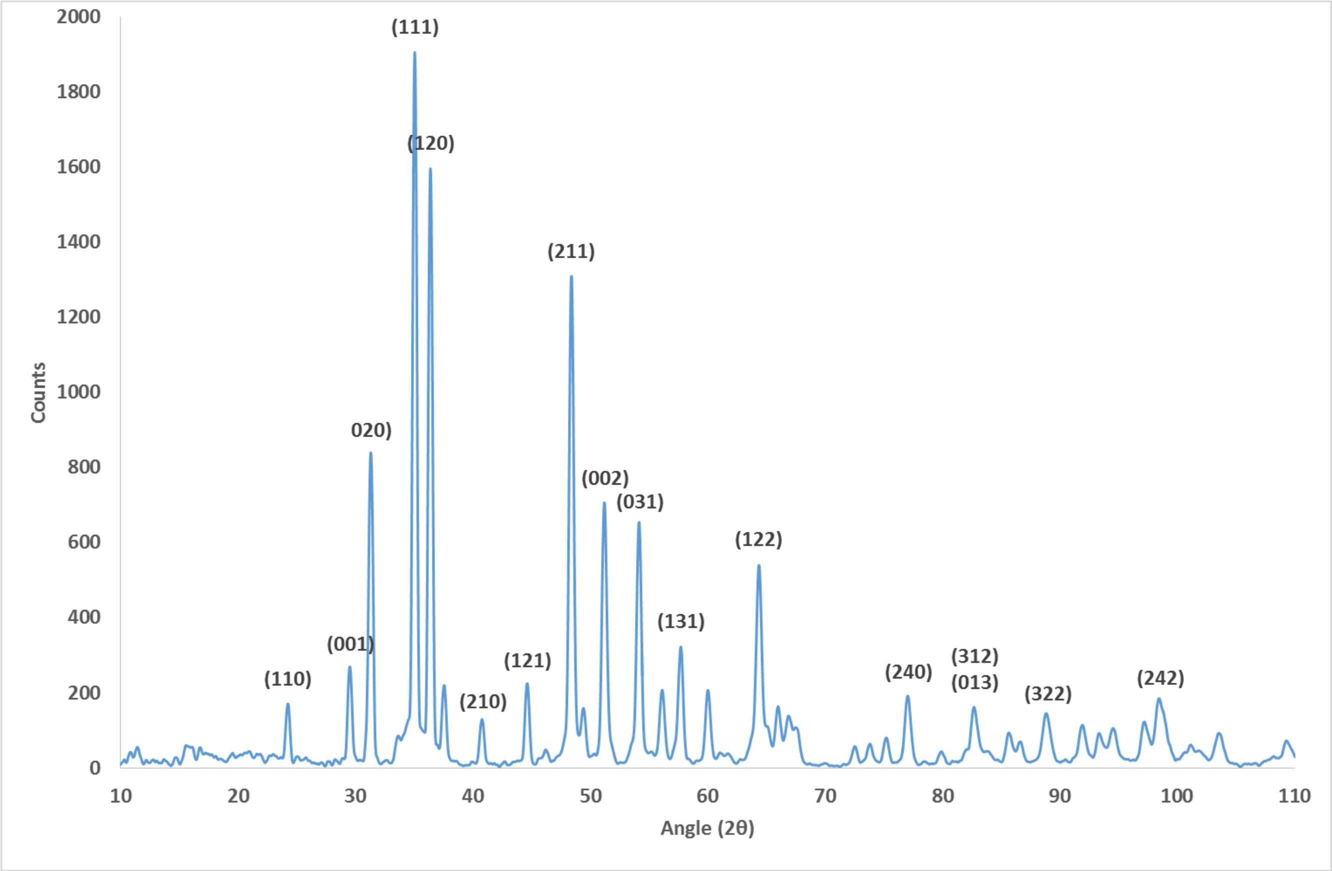

Figure: XRD pattern for $FeSe_2$ orthorhombic nanosheets

<u>**BISMUTH SELENIDE**</u>

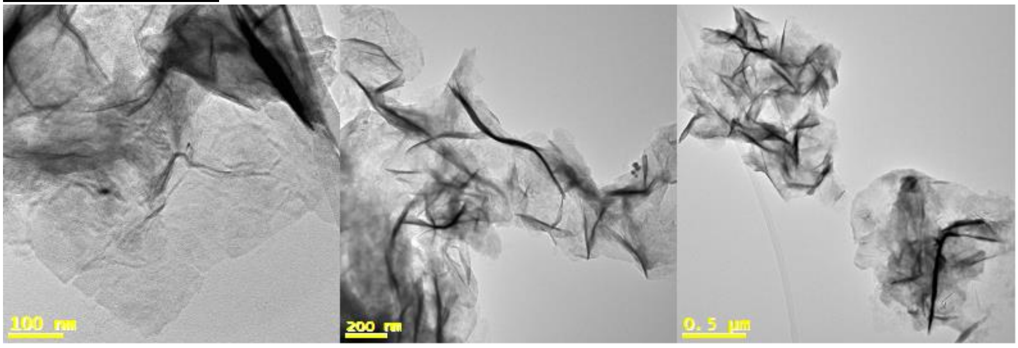

Figure: TEM images of bismuth selenide nanosheets

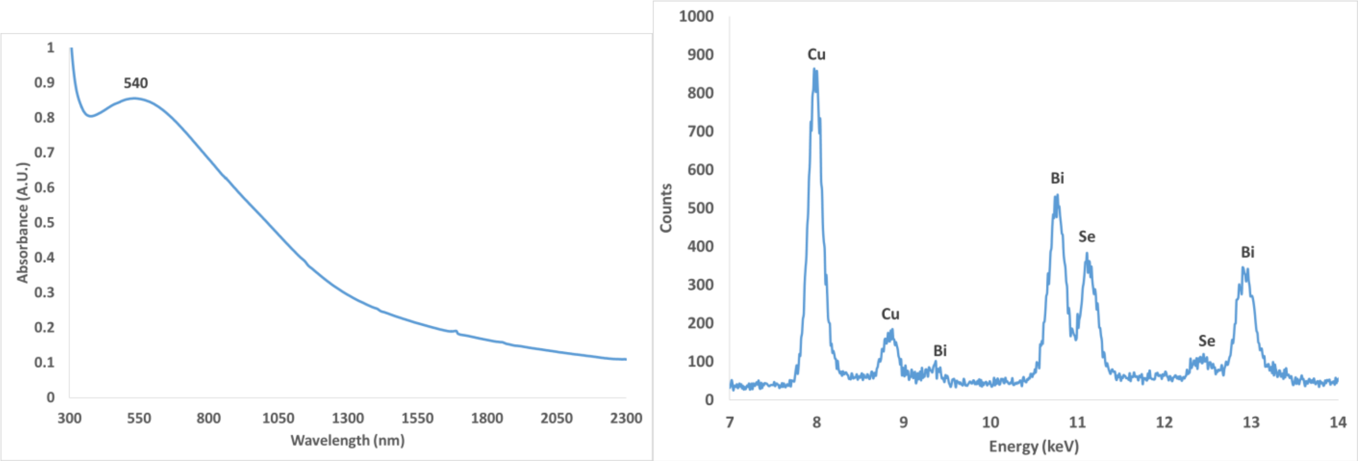

Figure: UV-IR absorption spectrum for bismuth selenide nanosheets (left), EDX spectrum for bismuth selenide nanosheets, showing a Bi/Se ratio of 55.5/44.5 (right)

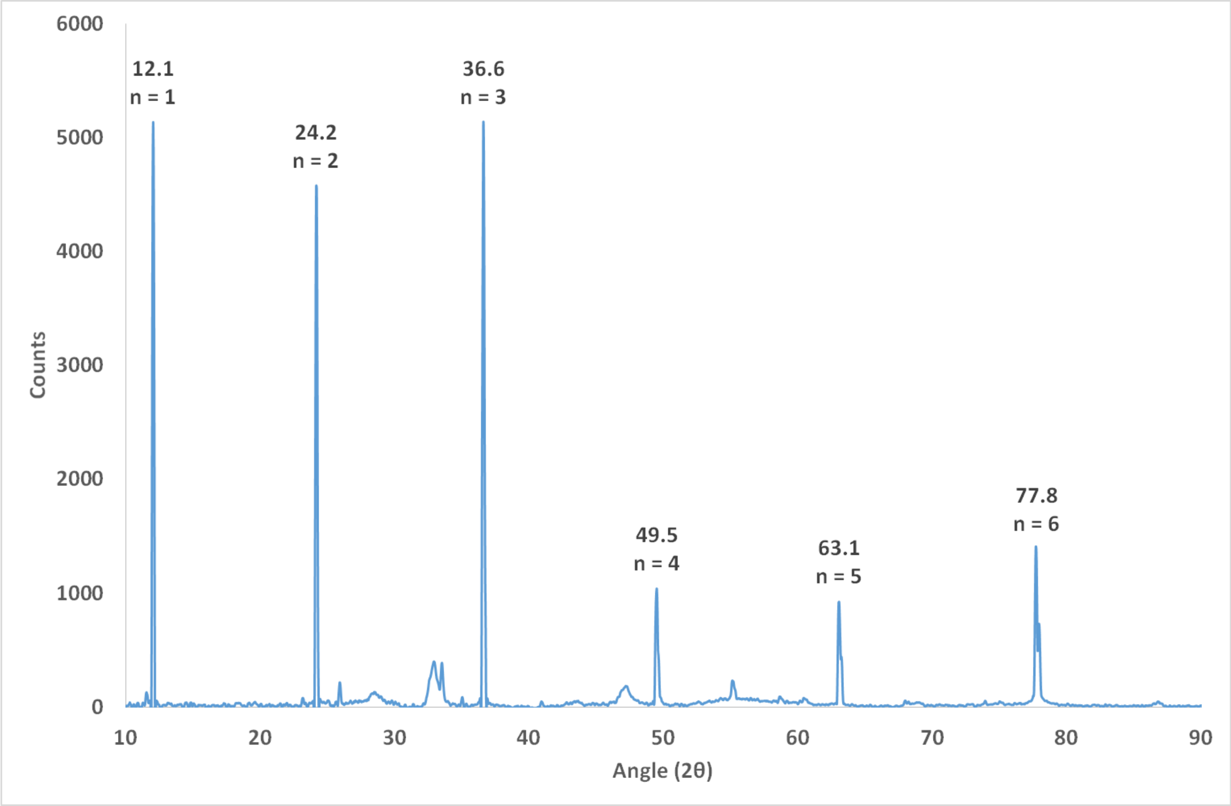

Figure: XRD pattern of stacked monolayer Bi_4Se_3 nanosheets with d = 0.735nm for $n\lambda = 2d\sin\theta$

OTHER PRODUCTS

NICKEL SULFIDE

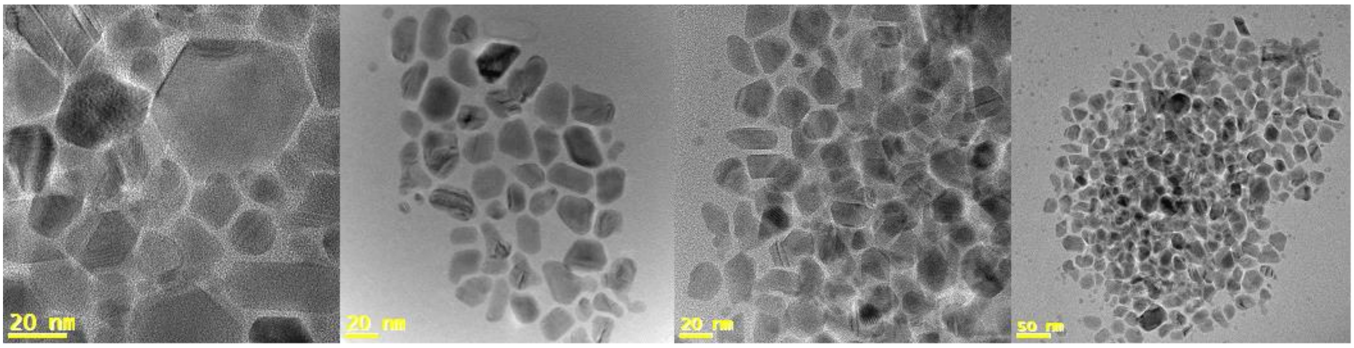

MOLYBDENUM SULFIDE

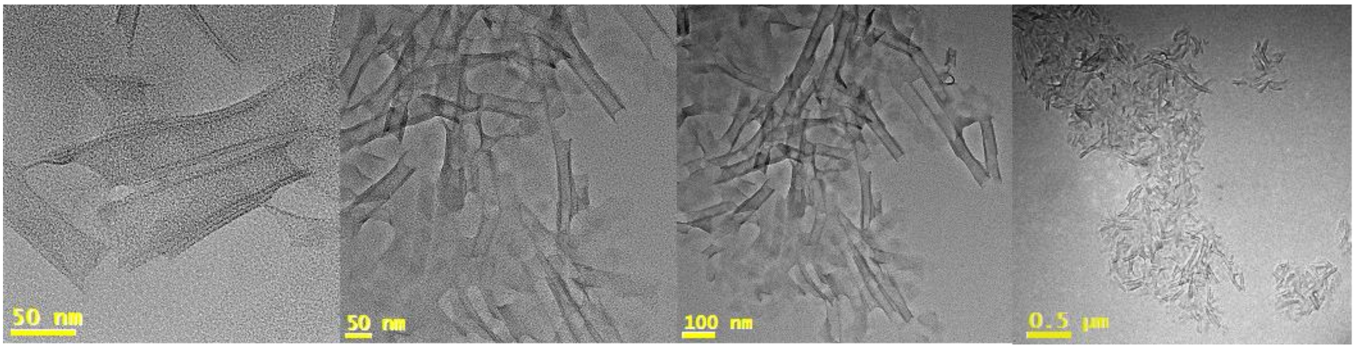

IRON SULFIDE

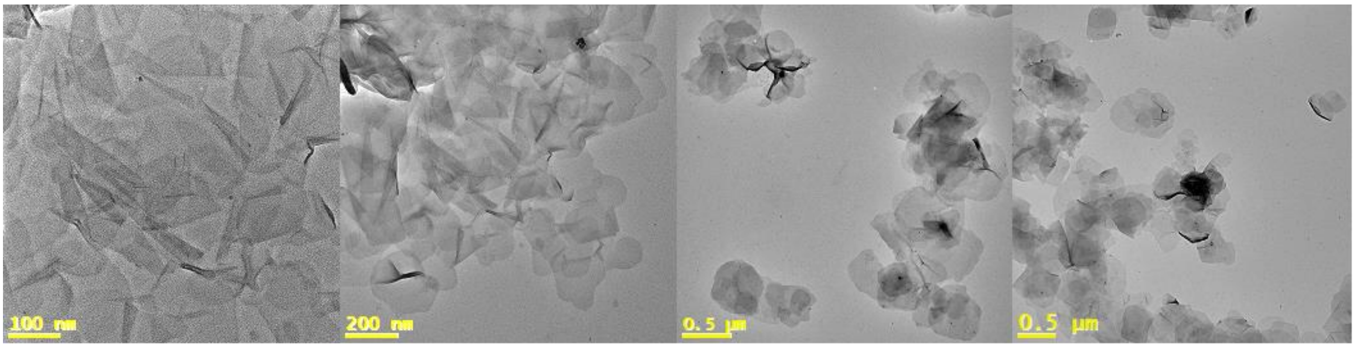

IRON SULFIDE

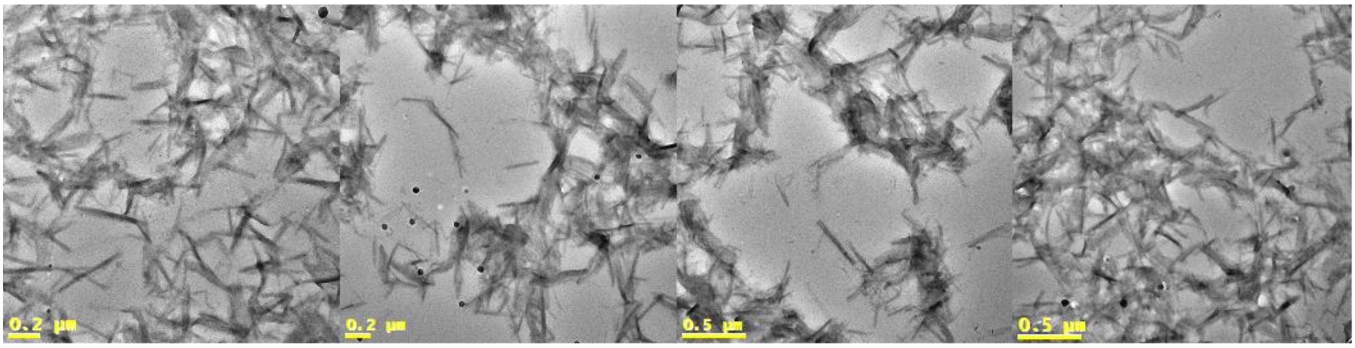

NICKEL SELENIDE

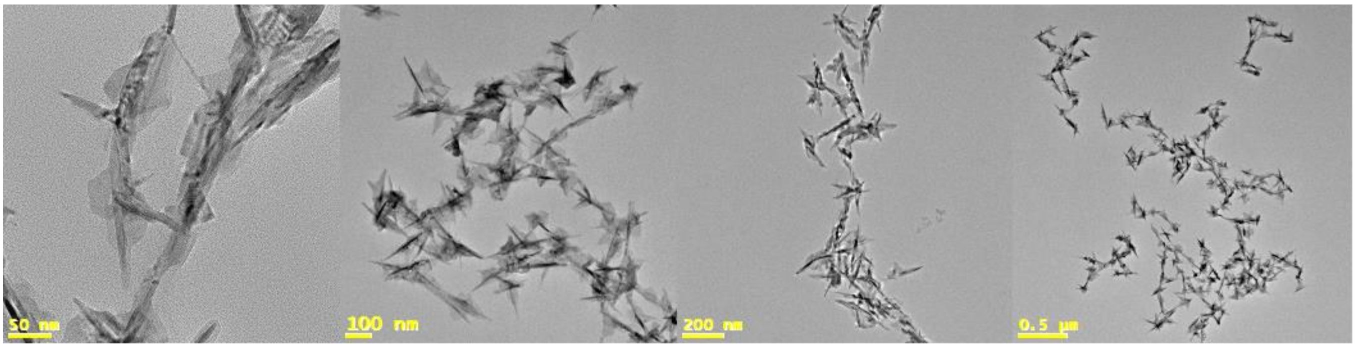

MAGNESIUM SELENIDE

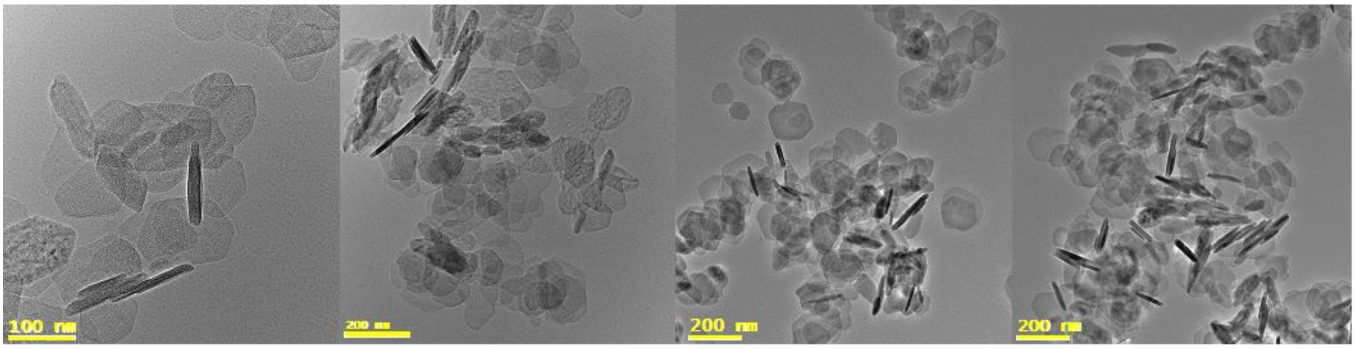

COPPER SELENIDE

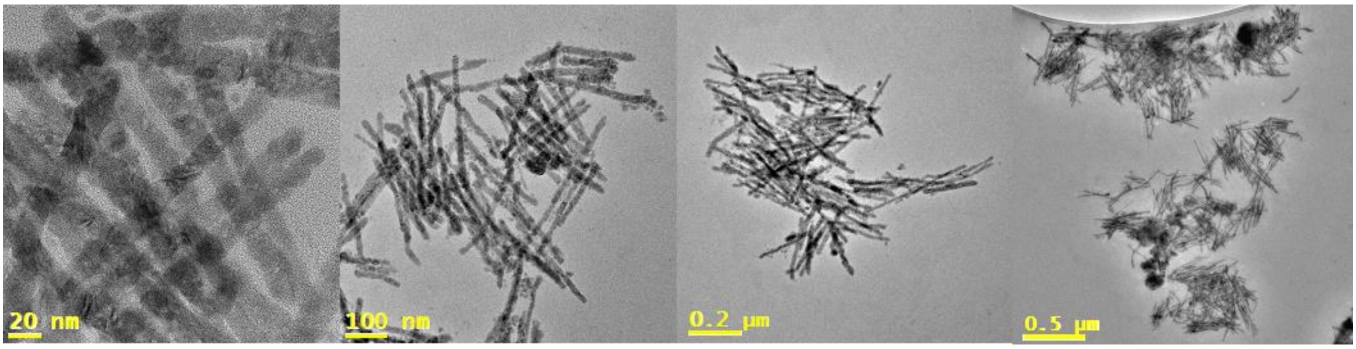

Supplement: Supplementary file 1 — Supplementary Information [file 41598_2018_21328_MOESM1_ESM.pdf]
